# Synthesis, Properties and Applications of Biodegradable Polymers Derived from Diols and Dicarboxylic Acids: From Polyesters to Poly(ester amide)s

**DOI:** 10.3390/ijms15057064

**Published:** 2014-04-25

**Authors:** Angélica Díaz, Ramaz Katsarava, Jordi Puiggalí

**Affiliations:** 1 Chemical Engineering Department, Polytechnic University of Catalonia, Av. Diagonal 647, E-08028 Barcelona, Spain; E-Mail: angelicadiaz07@hotmail.com; 2 Institute of Chemistry and Molecular Engineering, Agricultural University of Georgia, 13 km. David Aghmashenebeli Alley, Tbilisi 0159, Georgia

**Keywords:** poly(alkylene dicarboxylate)s, poly(ester amide)s, α-amino acid derivatives, bio-based polymers, carbohydrate derivatives, biodegradability, scaffolds, biomedical applications

## Abstract

Poly(alkylene dicarboxylate)s constitute a family of biodegradable polymers with increasing interest for both commodity and speciality applications. Most of these polymers can be prepared from biobased diols and dicarboxylic acids such as 1,4-butanediol, succinic acid and carbohydrates. This review provides a current status report concerning synthesis, biodegradation and applications of a series of polymers that cover a wide range of properties, namely, materials from elastomeric to rigid characteristics that are suitable for applications such as hydrogels, soft tissue engineering, drug delivery systems and liquid crystals. Finally, the incorporation of aromatic units and α-amino acids is considered since stiffness of molecular chains and intermolecular interactions can be drastically changed. In fact, poly(ester amide)s derived from naturally occurring amino acids offer great possibilities as biodegradable materials for biomedical applications which are also extensively discussed.

## 1. Introduction

Aliphatic polyesters are attracting increasing attention to solve “white pollution” concerns caused by traditional non-biodegradable polymers and also for their use as speciality polymers in applications that mainly involve the biomedical field [1–6]. In fact, polyesters are considered the most competitive biodegradable polymers commercialized up to now. Poly(alkylene dicarboxylate)s constitute a specific family of polyesters that offer great possibilities especially considering that most of them can be obtained from renewable resources. The extensive application of such polymers could not only mitigate the negative effect of non-degradable plastics on the environment but also reduce the dependence on fossil resources [7–10]. Poly(butylene succinate) (PBS) is probably nowadays the most significant poly(alkylene dicarboxylate) due to the unusual combination of good properties and the successful efforts carried out to solve typical problems of this family such as the limited molecular weight attained by polycondesation reactions between different monomers. Biodegradability of poly(alkylene dicarboxylate)s can be clearly improved by preparing random copolymers with low crystallinity and melting point, whereas incorporation of rigid units like biobased carbohydrates and even aromatic dicarboxylates may lead to better mechanical properties. Similar advantages can also be achieved through the incorporation of α-amino acids units since strong hydrogen bonding interactions are characteristic of the derived poly(ester amide)s, while degradation characteristics could be kept due to the susceptibility of formed ester and amide groups to enzymes with either estearase or protease activities.

The present work is focused to review the more recent developments performed with polymers derived from diol and dicarboxylic acids and highlights current activities on the biomedical field. The review is constituted by seven sections: the first five deal with generic issues concerning poly(alkylene dicarboxylate)s such as synthesis procedures, crystalline structure of homopolymers, characteristics of aliphatic copolymers. Incorporation of rigid units and degradability while the last two sections summarize more specific subjects such as selected applications and poly(ester amide)s based on α-amino acids.

## 2. Synthesis of Poly(alkylene dicarboxylate)s

### 2.1. Thermal Polycondensation

Aliphatic polyesters derived from diols and dicarboxylic acids are crystalline and biodegradable materials that can be used as an alternative of more conventional biodegradable polyesters prepared by ring opening polymerization of lactones (e.g., polyglycolide, polylactide and poly(ɛ-caprolactone)). Poly(alkylene dicarboxylate)s can be easily prepared by thermal polycondensation of diols and dicarboxylic acids or either their diesters or dichlorides. Nevertheless, this synthesis have problems associated to the use of solvents, and high vacuum and temperature conditions required to favour condensation reactions and the removal of water or alcohol byproducts. In general, the average molecular weight of poly(alkylene dicarboxylate)s is limited (*M*_n_ lower than 30,000 g/mol) if conventional polycondensation chemistry is used and toxic catalysts are avoided [11–13].

The use of dicarboxylic acid dichlorides is limited due to their high cost and occurrence of side reactions [14]. Nevertheless, high molecular weights were for example reported for poly(butylene adipate) (PBA) but under high vacuum and temperatures that render a high ratio of volatile byproducts [15,16].

### 2.2. Polycondensation Catalysts

Polycondensation of diols with dicarboxylic acids can also be performed by employing nonspecific lipases [17,18], but in this case final cost is also increased due to the expensive enzyme and even for the subsequent separation process that is required to eliminate the enzyme. Nevertheless, some advantages should be indicated since it is an eco-friendly method, that could proceed without any environmental pollution and effectively avoid both phase separation of the reactants and the use of toxic solvents. For example, the synthesis of poly(butylene sebacate) has been reported by a lipase-catalyzed synthesis in absence of organic solvents [19].

Polymerization of 1,4-butanediol with adipic or sebacic acid has successfully been carried out in diisopropyl ether using a lipase from *Mucor miehei* [20,21]. Nevertheless, the best results were achieved under solvent-free conditions with *Candida artarctica* (CALB) [22,23]. *Pseudomonas aeruginosa*, *Candida cylindracea* and *Klebsiella oxytota* lipases have also been revealed effective for the polycondesantion of sebacic acid with 1,8-octanediol in an aqueous medium [24].

The study of efficient catalysts in polyester polycondensation is another basic point to improve molecular weights. Titanium compounds (e.g., titanium (IV) isopropoxide, titanium (IV) isobutoxide or titanium (IV) *n*-butoxide) are usually employed to synthesize PBS since they display a high catalytic activity [25–27]. However, cautions should be taken into account since Ti-based catalysts may also enhance significant degradation reactions. Recently, new catalysts (e.g., those based on scandium triflates) have been developed to achieve the bulk polycondensation of methyl ester of sebacic acid with 1,4-butanediol under mild conditions (e.g., reaction temperature close to 35 °C) [28]. Moreover, these catalysts can be recovered and reused by solubilization in chloroform and extraction with water. Stannic components have also been studied for the synthesis of PBS in solution despite long reaction times being required [29]. The use of bismuth-based compounds for the synthesis of aliphatic polyesters has also been reported [30,31]. The efficiency of different catalyst has been just compared for PBS synthesis. Either organometal or metal-oxide compounds were considered, being elucidated the following metal ranking: Ti > Ge > Zr ~ Sn > Hf > Sb > Bi [32]. Thus, zirconium- and germanium-based catalysts appeared to be interesting substitutes for the commonly-used titanium compounds.

Mild condensation reactions can also be achieved by employing triflates (e.g., scandium triflate or lanthanide triflate) as catalysts as reported for the condensation of methylsuccinate with different diols [33,34] and sebacic acid with 1,4-butanediol [35]. Polycondensation of adipic acid or sebacic acid with aliphatic diols using an inorganic acid (H_3_PO_4_ or H_2_SO_4_) as catalyst combined with mild temperatures and low vacuum has also been evaluated [36]. This method was revealed effective to get aliphatic polyesters with appropriate molecular size (e.g., *M*_W_ close to 85,000 g/mol for sebacic acid derivatives) for possible use in the preparation of degradable disposable medical supplies.

### 2.3. Ring Opening Polymerization Methods

Ring opening polymerization (ROP) has several advantages over polycondensation, but the low availability and high manufacturing costs of cyclic ester oligomers (CEOs) at industrial scale has prevented its general application for the synthesis of poly(alkylene dicarboxylate)s. Nevertheless, several efforts have been focused to develop CEOs by enzymatic synthesis since mild reaction conditions are usually required and high selectivity is obtained. The enzymatic cyclization of α,ω-diacids (C_4_–C_14_) and diols (C_7_–C_18_) with lipases of different origin (e.g., CALB) has been studied under different conditions, being found that macrocycles were favoured when high dilution, high temperature and polar solvents were employed [37].

The synthesis of PBS starting from cyclic butylene succinate oligomers via ROP using CALB as biocatalyst has also recently been reported [38]. The enzyme-synthesized PBS product had an average molecular weight of *M*_W_ 130,000 g/mol that was significantly higher than that obtained by the direct polycondensation of 1,4-butanediol (BDO) and dimethyl succinate by lipase catalysis (*M*_W_ of 45,000 g/mol). It has also been indicated that CEOs as an unique family of products can be obtained by CALB-catalyzed esterification of a non-activated succinic acid (SA)/BDO mixture under water removal conditions and in a fed-batch system [39].

### 2.4. Poly(alkylene dicarboxylate)s from Biobased Monomers

Biodegradable poly(alkylene dicarboxylate)s derived from biobased monomers have an additional interest as above indicated and specifically those obtained from diols like 1,3-propanediol, BDO and 1,10-decanediol, and dicarboxylic acids such as those based on carbohydrates and SA, adipic acid, sebacic acid and fatty acids. Thus, SA and BDO can be fully synthesized using sugar-based feedstock by bacterial fermentation [40,41]. Specifically, SA can be obtained with high yield by *A. succiniciproducens* using glycerol as substrate^3^ whereas BDO can be easily prepared by the reduction of SA. The indicated monomers are employed to get a fully bio-based PBS, which is one of the most investigated degradable polyesters because of its relatively high melting point (*T*_m_ = 103 °C) and favorable mechanical properties. In fact PBS could be a very interesting substitution for polyolefins in some applications.

A number of promising approaches toward bio-based production of adipic acid, or more often, precursors of adipic acid like *cis*-muconic acid and glucaric acid, have nowadays been described, offering alternatives to the established petroleum-based processes [42]. In general, procedures combine a biotechnological process for fermentative production of a bio-based precursor using selected or engineered microbial strains and an environmentally conscious chemo-catalytic process for the conversion of the precursor to adipic acid. Large efforts are focused to get non-food-based fermentable raw materials, in particular lignocellulosics, by the degradation of structural carbohydrates of plant cells [43]. Bio-based production of adipic acid could also be achieved with engineered yeast strains by α- and/or ω-oxidation of long-chain *n*-alkanes, alcohols or fatty acid substrates [44].

1,3-Propanediol (1,3-PD) is a chemical compound that can be obtained from several bioprocess cultivation techniques facilitated by natural and/or genetically engineered microbes [45]. 1,3-PD is produced in nature by bioconversion of glycerol but also the development of recombinant strains makes feasible its production from sugars like glucose. 1,3-PD has gained interest as a raw material for biodegradable polyesters due also to the presence of odd number of methylene units. Homopolyesters and copolyesters constituted by 1,3-PD and carboxylic acids can be easily synthesized by two-step polycondensation in the bulk at high temperature and under reduced pressure [46].

Polyesters having long linear polymethylenic segments are gaining interest since crystallinity may be enhanced as well as their crystallization and melting temperatures. Fatty acids coming from plant oils have extensively been considered and specifically C_19_ and C_23_ α,ω-dicarboxylic acid esters have been prepared from methyl oleate and ethyl erucate, respectively [47,48].

**Figure 1.**
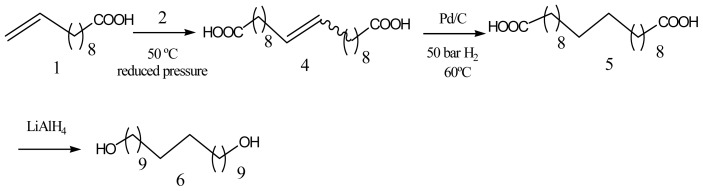
Synthesis of monomers of polyester 20,20, based on [50].

Long chain and even number α,ω-dicarboxylic acid esters can also be obtained from metathesis reactions by coupling of two CH_2_=CH(CH_2_)_n_COOR fragments, followed by hydrogenation to the saturated product. These ω-functionalized 1-olefins can be prepared by pyrolysis of fatty acids. Thus, ricinoleic acid gives rise to undecenoic acid [49], which after the metathesis reaction renders the 1,20-eicosanedioic acid. This monomer was successfully polymerized with eisosane-1,20-diol, obtained from the diacid via reduction (Figure 1), to yield the semicrystalline polyester 20,20 (*χ*_c_ = 68%, *T*_m_ = 106 °C and Δ*H*_m_ = 151 J/g) [50].

Saturated aliphatic dicarboxylic acids with chain lengths from C11 to C18 can be produced via fermentation of a vegetable oil with genetically optimized yeast strains (e.g., *C. tropicalis* with a blocked β-oxidation) [51]. This biotechnological synthesis provides a cost-efficient and green alternative process to get new biodegradable polyesters. For example, biobased ω-carboxy fatty acid monomers like 1,18-*cis*-9-octadecenedioic, 1,22-*cis*-9-docosenedioic and 1,18-*cis*-9,10-epoxy-octadecanedioic acids were obtained by mutated *C. tropicalis*, as building blocks for lipase-catalysed conversion to linear unsaturated and epoxidized polyesters. These functionalyzed polymers are interesting since allows further cross-linking reactions and also the incorporation of bioactive moieties [44].

Polyalkylene dicarboxylates containing rigid units merit attention since an improvement of mechanical and thermal properties could be expected after their incorporation in the main chain. Specifically, aliphatic-aromatic copolymers have been deeply studied as will then be described, but polymers incorporating 1,4-cyclohexylene units are also worthy to be mentioned. Specifically, derivatives of 1,4-cyclohexanedimethanol, 1,4-cyclohexanedicarboxylic acid and dimethyl-1,4-cyclohexanedicarboxylate have been considered [52]. These monomers have the advantage that can be prepared from biomass (e.g., from terpenes) [52,53], although costs are presently too much high for a commercial application. The indicated cycloaliphatic ring monomers were stable and able to react in two-step polycondensation without particular problems. The combination of specific cyclic building blocks with the correct *cis*/*trans* ratio makes feasible to get polymers with tunable properties. Thus, the effect on thermal properties reached its maximum level when the cycle-containing monomers were the diacid or the diester and when the *trans* isomers predominated [53].

Isosorbide and isomannide are 1,4:3,6-dianhydrohexitols (DAHs) derived from d-glucitol and d-mannitol, respectively. These carbohydrates are obtained from polysaccharides such as starch, cellulose, and mannans extracted from cereals [54]. DAHs have been employed as biobased building blocks for the synthesis of polyesters with liquid crystal and biodegradable properties [55–58]. The chirality in the DAHs is a fundamental parameter for modulation of polyesters properties based on them [54].

Syntheses of DAHs derived polyesters are mainly performed in the melt or in solution using activated diacid components like acid chlorides. Polymerization at high temperatures may lead to an undesirable triol moiety due to the generation of HCl that can attack the cyclic ethers present in the monomer unit. The use of biobased dicarboxylic acids such as SA to prepare isosorbide derivatives has also been tested. Bulk polycondensation rendered samples with relatively low molecular weight and high glass transition temperature (*i.e.*, higher than 48 °C) due to the chain rigidity imposed by the cyclic structure of isosorbide [59]. A series of polyester based on isosorbide was also synthesized from succinic, adipic, and sebacic acids via simple nonsolvent polycondensation [60]. Polymers supported cell growth and showed versatile properties, including *T*_g_, *T*_m_, biodegradability and mechanical strength.

Hyperbranched (HB) polymers are materials that appear suitable for a wide range of applications (e.g., surface modification, reactive component in coating and resin formulations, nanotechnology, polymeric plasticizers and polymeric additives for improving rheology and flow) due to the excellent properties (e.g., high solubility, low solution viscosity, melt rheology characteristics and high functionality) given by the high branching and terminal groups densities [61–63]. HB polymers can be prepared in a one-pot synthesis since in general a strict control on branching and the final structure is not necessary. Nevertheless, HB polymers have some limitations that concern the high branching density achieved when branching sites exist in each repeating unit. In this case, formation of molecular entanglements in the bulk material is prevented and high brittleness and poor mechanical properties are expected. The use of linear oligomeric starting materials should increase the distance between branching points and potentially render hyperbranched hybrid materials with better characteristics.

**Figure 2.**
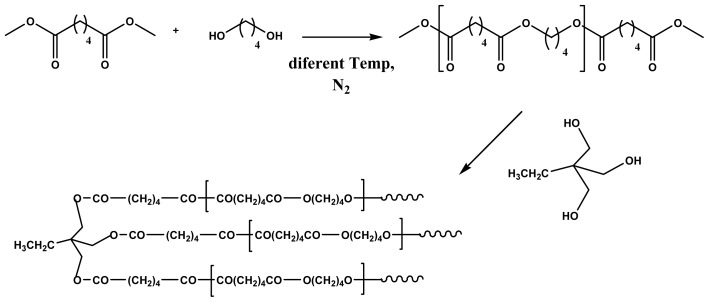
Synthesis of linear-hyperbranched hybrid poly(butylene adipate) copolymers, based on [65].

Specifically, biodegradable polyester hybrids have been synthesized by melt polycondensation of PBA prepolymers terminated with carboxylic acid groups with glycerol or pentaerythritol as branching agent [11]. An accurate study on the synthesis and characterization has also been performed for the one-pot reaction between tailored PBA samples terminated with methyl ester groups and 1,1,1-tris(hydroxy methyl)propane (TMP) [64]. This synthesis involved two steps as depicted in Figure 2. It was found that both inter- and intramolecular transetherification occurred as the mol % of TMP and the reaction times increased, whereas at low mol % of TMP (*i.e.*, 1.5 mol %) and low reaction time the transesterification reaction involving the hydroxyl groups of TMP and the methyl ester end groups of the PBA prevailed. Both intermolecular side reactions (transetherification and transesterification) could lead to a molecular weight increase.

PBS has a linear structure that may lead to low melt strength and melt viscosity. In this case applications in thermoplastic processing, foaming, and filming may be hindered, being highly interesting to enhance molecular weight by employing chain extenders and also by introducing branches into the chain structure. These derivatives have been commercialized as Bionolle^®^, a material with an excellent processability that can be employed in the fields of textile and plastics [65,66].

Octamethylcyclotetrasilazane, hexaphenylcyclotrisilazane and 2,2′-(1,4-phenylene)-bis(2-oxazoline) have been proved highly effective as chain extenders [67,68], especially when catalysts like *p*-toluenesulfonic acid were employed. However, transesterification reactions may also take place during the chain extension reaction causing disturbing effects on the chain microstructure.

**Figure 3.**
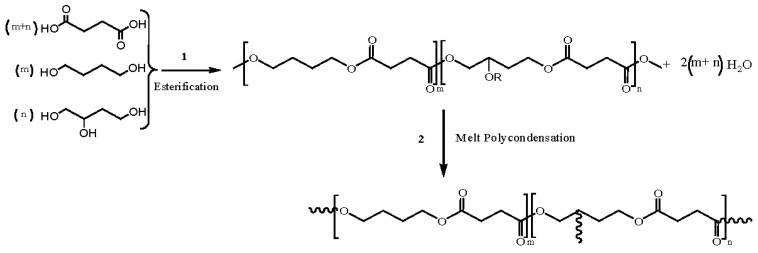
Synthesis of long-chain branched poly(butylene succinate) (PBS), based on [72]. The R group means the result of esterification with fragments having carboxylic end groups.

Incorporation of long-chain branches can be easily performed by adding multifunctional comonomer branching agents during the polycondensation reaction. Entanglement among polymer chains in melt and concentrated solutions should logically be increased with the presence of long-chain branches and an improvement of rheological properties should be expected [69,70]. TMP and 1,2,4-butanetriol (1,2,4-BT) have been employed as chain branching agents in the synthesis of PBS, being reported to render a significant increase of complex viscosities, while a decrease of loss tangent or elongation at break was also indicated [71,72]. Addition of only 1 mol % of 1,2,4-BT segments (Figure 3) has a great impact on mechanical properties and appears for example sufficient to enhance the tensile strength of linear PBS by 31% [71].

The effect of ethyl and *n*-octyl branches on the properties of PBS have also been studied [73,74] being reported in this case that these relatively short branches may cause an improvement of biodegradability, the elongation at break, and the tear strength without notable decrease of tensile strength and tensile modulus. However, the melt viscosity of these branched PBS copolymers was even lower than that of linear PBS.

A widespread application of PBS is also prevented due to its poorer mechanical properties and higher price compared to conventional polyesters based on aromatic dicarboxylate units such as poly(ethylene terephthalate) and poly(butylene terephthalate) [75]. These units should reduce the final cost of the polymer and on the other hand may provide a good combination of physical properties. Different approaches have been undertaken to incorporate aromatic units to PBS, although biodegradability should be in general negatively affected [75,76].

## 3. Crystalline Structure of Poly(alkylene dicarboxylate)s

Structural studies on polyalkylene dicarboxylates indicate variability on the molecular conformation. Kink conformations can be found in polymers with a low number of methylene groups, such as polyesters 2,4, 2,6, 2,8, 4,4, 4,6 and 6,6 [77–82]. In fact, quantum mechanical calculations on small diesters [83] showed the tendency of methylene units to adopt gauche conformations when a short aliphatic segment was placed between two carbonyl groups. On the other hand, an extended conformation was postulated for polyesters with long polymethylene sequences [84]. In general, all the above referred polyesters crystallize according to monoclinic or orthorhombic unit cells [85], the dimensions of the chain axis projected unit cell being similar to that reported for polyethylene when the polyester has an extended conformation. In this case, energy calculations suggested a packing structure where molecular chains had an orientation of methylene segments similar to that of polyethylene (*i.e.*, a setting orientation angle close to ±45°) [86].

A glide plane *a* has usually been postulated for relating the two molecules with an extended conformation that belongs to the unit cell. Consequently, molecular chain segments were not shifted along the chain axis direction when they pack according to orthorhombic unit cells, and models were built by placing the ester groups of the different chains at the same level. Nevertheless, structural studies carried out with PBS [87], poly(hexamethylene sebacate) [88] and poly(dodecamethylene sebacate) [13] demonstrated that the two molecular chains of the unit cell were related by a diagonal glide plane *n*. This packing symmetry may have important repercussions on the lamellar folding surface. Specifically for polyester 12,10, folds constituted by methylene groups were postulated to occur along the (100) crystallographic direction where chain segments were not shifted, whereas ester groups were supposed to be involved along the (110) direction where neighbouring chains had a *c*/2 shift [89].

**Figure 4.**
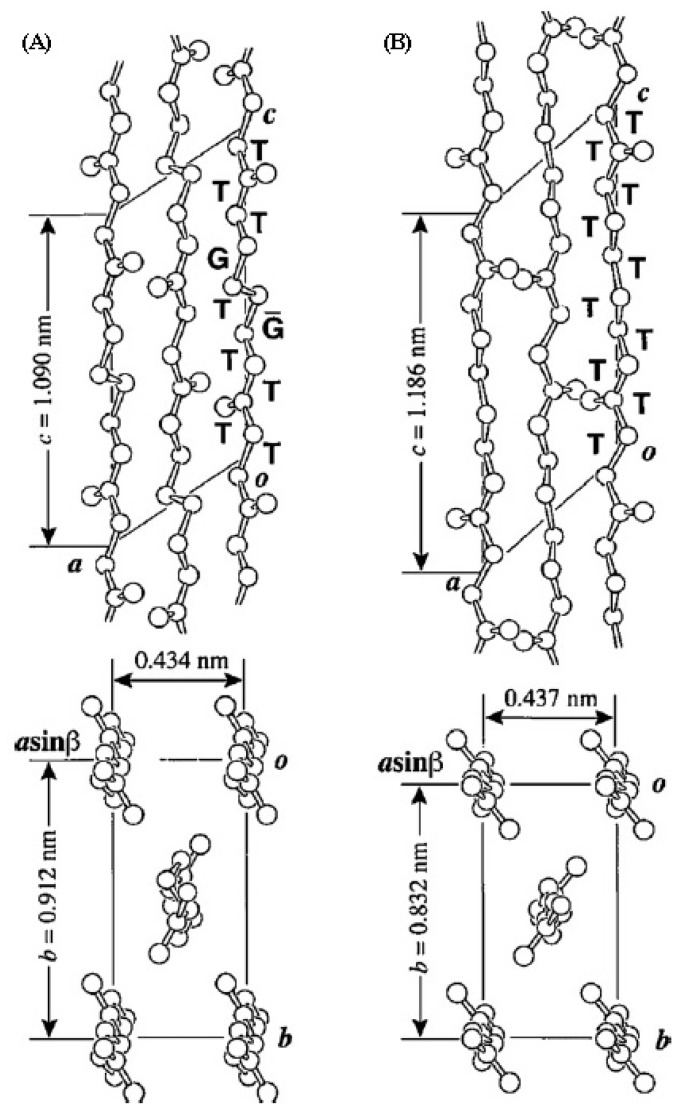
Crystal structures for the α-form (**A**) and the β-form (**B**) of PBS on the *a–b* (bottom) and *b–c* (top) base planes. All hydrogen atoms are omitted. Reproduced from Ichikawa *et al.* [87], copyright (2000), by permission of Elsevier Science Ltd.

Polymorphism has been determined for some polyesters having short polymethylene sequences (*i.e.*, poly(ethylene succinate), PBS and poly(butylene adipate)) [90] since an extended conformation (β-form) was found in addition to the indicated kink conformation (α-form) (Figure 4). In general, the transition from the α-form to the β-form occurred reversibly under the application and release of tension.

**Figure 5.**
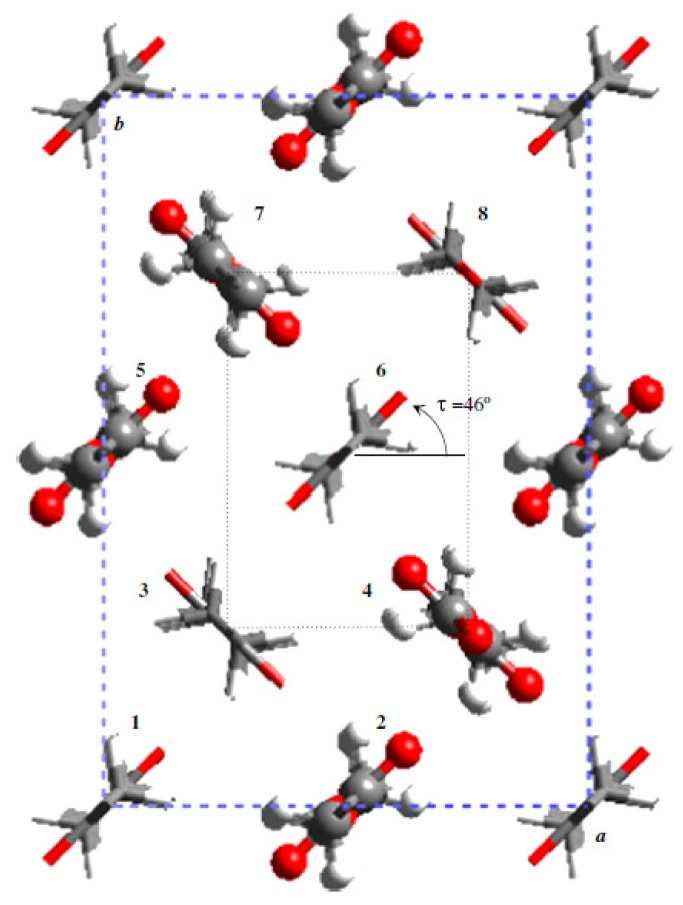
View parallel to the *c*-axis direction showing the packing of polyester 6,4 assuming a large unit cell with *a* and *b* parameters of 1.612 and 1.464 nm, respectively. In projection this packing can be defined by a conventional rectangular cell containing only two chain segments (dotted lines). Reproduced from Gestí *et al.* [94], copyright (2007), by permission of Elsevier Science Ltd.

Electron diffraction patterns of polyesters with the extended zig-zag conformation indicated that the unit cells projected along the chain axis were rectangular, the parameters being close to *a* = 0.500–0.504 nm, and *b* = 0.73–0.75 nm. These cells contained as indicated two molecular segments, but recent works on some BDO (*i.e.*, polyesters 4,8, 4,10 and 4,12) [91] and 1,6-hexanediol (*i.e.*, polyesters 6,8 and 6,12) [92] derivatives have revealed the existence of unit cells where the *b*-axis parameter was doubled. These more complex structures were characterized by the fact that neighbouring molecules along the *b*-axis were only equivalent in chain axis projection. Complex structures were also determined for polyesters 6,6 [93] and 6,4 [94] (Figure 5) since the corresponding unit cells contained eight molecular segments.

## 4. Copolymers Constituted by Different Diol or/and Dicarboxylic Acid Units

Preparation of new copolyalkylene dicarboxylates offers a key opportunity to increase the range of degradable materials and even to generate a set of products with easily tunable properties. Thus, different types of PBS copolymers and blends have recently been developed as above explained to increase biodegradability, decrease costs, increase commercial offer and even modify final properties.

Incorporation of comonomers has also a great influence on melting behavior, sample crystallinity, lamellar surface morphology, and consequently enzymatic degradability [95–97]. Several works have recently been addressed to study the crystallization process of random copoly(alkylene dicarboxylate)s. This kind of studies is significant not only because of the processability and applicability of the materials, but also for its strong correlation with the biodegradation process [98]. The studied copolymers were mainly constituted by even units, and specifically poly(butylene succinate-*co*-ethylene succinate) [99], poly(hexylene succinate-*co*-hexylene adipate) [100], poly(hexylene sebacate-*co*-hexylene adipate) [101,102], poly(hexylene sebacate-*co*-hexylene suberate) [9] and poly(hexylene suberate-*co*-hexylene adipate) [103] are representative examples.

Random copolymers constituted by two crystallizable units may show a different crystallization behavior depending on the compatibility of the two components in crystal lattices. Isomorphic *co*-crystallization is characterized by the formation of only one crystalline phase containing both crystalline units. In this case, these units must meet strict molecular requirements such as having a similar chemical structure and even molecular conformation to allow their incorporation into the resulting crystalline structure with minimum distortion. In contrast, two crystalline phases and pseudo-eutectic behavior are observed when isodimorphism occurs. Increase of minor comonomer concentration in each crystalline phase lowers the melting temperature and crystallinity of copolymers. Obviously, requirements are in this case less strict, and consequently isodimorphism is most commonly observed in random copolymer crystallization.

**Figure 6.**
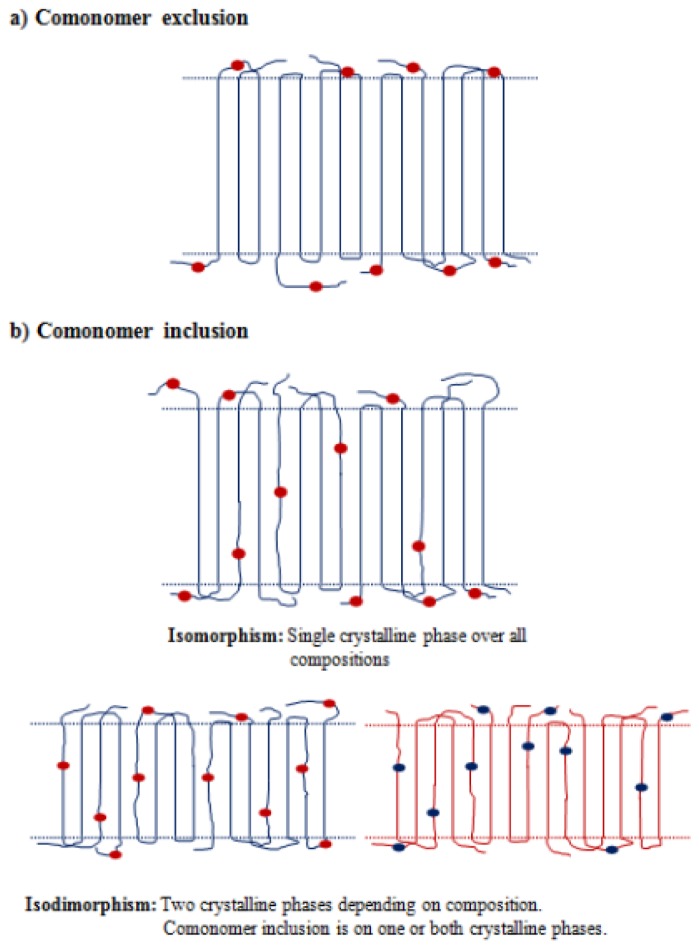
Scheme of the comonomer (**a**) and inclusion (**b**) arrangements in lamellae for full exclusion and inclusion models. In the latter case, isomorphic and isodimorphic structuresare considered. In the last case, the two crystalline phases are represented with blue and red colors and the comonomer inclusion has been drawn for both phases. Reproduced from Díaz *et al.* [104], copyright (2014), by permission of Elsevier Science Ltd.

Isodimorphism implies that at least one of the two crystalline phases incorporates the corresponding minor component in its crystalline lattice. In this sense, distribution of comonomer units in crystalline and non-crystalline regions is another differentiating factor in semicrystalline copolymers consisting of a random distribution of two chemical units. Comonomer units can generally be excluded in the organized lamellae and remain only in the amorphous phase or are compatible in such a way that they are able to share a crystalline lattice (Figure 6).

For example, poly(butylene azelate-*co*-butylene succinate) copolymers clearly showed a melting point depression with comonomer content, which fitted exclusion models [104]. Combined DSC and FTIR data demonstrated a peculiar behavior for these copolymers when had a similar azelate/succinate content since crystallization of butylene azelate rich fragments was enhanced by rapid cooling [105]. It was postulated that the development of the poly(butylene azelate) crystalline phase was more favored when the temperature associated with the formation of its primary nuclei was reached fast enough to avoid termination of previous PBS crystallization. Probably, crystallization of poly(butylene azelate) was hindered in a confined system derived for a complete PBS crystallization.

## 5. Biodegradable Poly(alkylene dicarboxylate)s Having Rigid Aromatic or Carbohydrate Units

Incorporation of aromatic moieties allows increasing the strength and performance properties of biodegradable aliphatic polyesters. Thus, these aliphatic-aromatic polymers appear an ideal solution to get fully biodegradable materials with properties similar to commodity polymers such as polyethylene. Nevertheless, the use of these aromatic derivatives as biodegradable materials may be restricted in some cases by legislation when a minimum content from renewable resources should be required.

**Figure 7.**
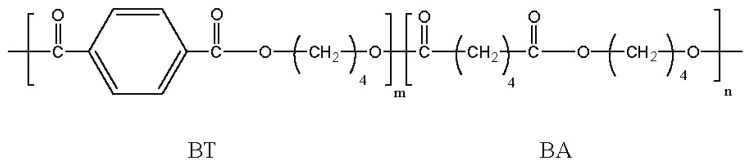
Chemical structure of poly(butylene adipate-*co*-terephthalate) showing the rigid and flexible moieties derived from terephthalic and adipic units, respectively.

Ecoflex™ and Eastar Bio™ produced by BASF and Eastman, respectively, are probably the most important commercially available aliphatic-aromatic polyesters. These copolyesters are derived from 1,4-butanediol and adipic and terephthalic acids that render soft and hard segments, respectively (Figure 7). Metabolization of this kind of copolyesters was proven in a compost environment [106] and even thermophilic actinomycetes microorganisms (e.g., *Thermomonospora fusca* strain) were found capable to rapidly depolymerize these copolyesters [107,108], being the degrading enzyme characterized as a lipase-like hydrolase [109].

Random copolyesters based on BDO and different ratios between adipic and terephthalic units (PBAT) were synthesized from thermal polycondensation of the appropriate mixture of monomers or by melt transesterification of the mixture of homopolymers [110]. Hydrolytic degradation was demonstrated to took mainly place through cleavage of the adipate ester groups. Therefore significant differences in the degradation rate were found depending on the adipate content. Copolymers appeared to be enzymatically degradable with lipases (e.g., from *Pseudomona cepacia*), although the degradation rate was slow and again dependent on the adipate content.

PBAT copolymers exhibit a highly complex micro-structure since well differentiated domains exists side by side in one polymer chain as a consequence of the highly different aliphatic and aromatic monomers. Further investigations on the degradation process were carried out from well characterized aliphatic–aromatic polyesters (*i.e.*, blend, block, random and alternating samples having a 50:50 ratio between adipic and terephthalic units) that were exposed to degradation tests using a lipase as biological active agent [111]. Results clearly demonstrated that the mobility of the polymer chains (the ability of chain segments to temporarily escape for a certain distance from the embedding crystal) was the major and general controlling factor for the biodegradability of polyesters. The mobility of the polymer chains in the crystalline regions at the temperature at which degradation is evaluated depends on how higher is the melting temperature: the susceptibility to an enzymatic attack is significantly high when the melting point of the crystalline domains is not more than 30–40 °C above the temperature of degradation, and decreases for higher melting points.

Biodegradation of a series of aliphatic-aromatic copolyesters derived from terephthalic acid, BDO, and suberic acid / sebacic acid have also been investigated. Results pointed out a determinant influence of crystallinity on degradation rate [112].

Cocrystallization could occur in the aliphatic-aromatic copolyesters derived from adipic and terephthalic acids since the soft BA segments could adjust their chain conformation to that of the rigid BT segments [113]. During biodegradation the crystallinity of copolymers clearly increased as expected for a faster degradation of amorphous regions.

**Figure 8.**
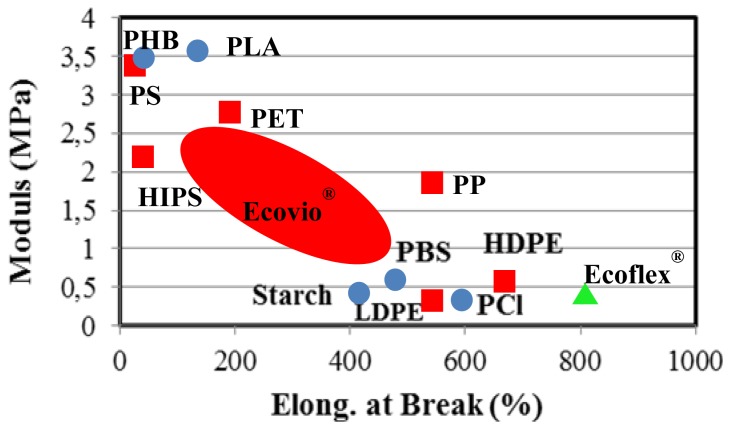
Comparison between mechanical properties of Ecoflex™, Ecovio™ and representative commodity polymers.

Blending of polymers is a practical way to obtain a material with tailored properties. Hence, Ecoflex has been blended with polylactide to provide a fully degradable material (Ecovio™) with significant renewable raw content and good mechanical properties (Figure 8). Blends of polyhydroxy(butyrate-*co*-valerate) (PHBV) and PBAT have recently attracted interests in research environment, since can also provide an excellent combination of mechanical properties of each component [114]. Incorporation of low-cost biomass into PHBV/PBAT blend to produce less expensive biocomposites has been considered and specifically promising results were attained with biofuel coproducts [115].

Interestingly, the addition of saturated fatty acids (e.g., caproic, lauric and stearic) was found to increase the crystallinity of biodegradable films made from blends of starch, glycerol and PBAT. Hydrophilicity of films did not decrease by addition of the fatty acid, which played a higher influence on crystallinity as longer the carbon chain was [116].

Agar is a well-known biopolymer constituted by 1,3-linked β-d-galactopyranose and 1,4-linked 3,6-anhydro-α-l-galactopyranose residues that has been widely used in different reinforcing agents for PABT [117]. These biocomposites were developed using extrusion and injection molding technique and physicomechanical, thermal, and morphological analyses performed.

Analysis of nano-biocomposites based on PBAT and organomodified layered silicates has also been undertaken [118,119]. It has been concluded that the appropriate incorporation of montmorillonite as a nanofiller can improve PBAT properties (e.g., enhanced thermal stability and increased stiffness) and thus increase the attractiveness of the polymer as sustainable material.

Poly(butylene 1,12-dodecanedioate) (PBD) together with other polyesters derived from the 1,12-dodecanedioic acid are highly promising materials due to their high thermal stability, high level of crystallinity, and high crystallization rate [120,121]. Therefore, novel aliphatic-aromatic random copolyesters based on the incorporation of terephthalic acid units to PBD have received attention [122]. A fine modulation of the content of the co-units for this class of copolymers was required in order to achieve materials with the most desired properties in terms of physical performances and biodegradability since as above indicated these properties are negatively correlated.

Copolymers having PBS and poly(1,2-propylene terephthalate) (PPT) blocks were synthesized via chain extension from a mixture of diol terminated prepolymers using hexamethylene diisocyanate as a chain extender. The amorphous and aromatic PPT segment was chosen to modify the mechanical properties of PBS and reduce its cost. It was found that the incorporation of appropriate amounts of PPT segments improved the impact strength of the multiblock copolymers without seriously decreasing tensile strength, flexural strength, thermal stability and melting point. These good properties were a direct consequence of the presence of amorphous and rigid PPT blocks, the establishment of allophanate crosslinks and a regular sequential structure. Crystallization rate and biodegradation rate were reduced by the copolymerization but remained at acceptable values when the PPT content was 5 wt. % [123].

Aliphatic units derived from naturally occurring carbohydrates (e.g., tetroses, pentoses and aldoses) have been employed to impart degradability and hydrophilicity to aromatic polyesters (e.g., poly(butylene terephthalate) and poly(ethylene terephthalate)) [124–128]. Thermal properties of these copolymers were found very dependent on the sugar constitution and copolyester composition. In general, samples were thermally stable above 300 °C and semicrystalline for contents in carbohydrates up to 30%.

Incorporation of bifunctional, sugar-based monomers with a bicyclic structure is of great interest, due to their ability to add stiffness to a polymer backbone. High molecular weight polycondensates containing such monomers can be prepared by solid-state modification (SSM) of an existing semicrystalline polyester. SSM is performed below the melting temperature of the crystalline phase of the polymer and far above its glass transition temperature. In this way, chain segments present in the mobile amorphous fraction are modified with the added (macro)monomer by transesterification reactions while mobility restrictions prevent the crystalline phase as well as the rigid amorphous fraction from taking part in transesterification reactions [129].

Macrodiols derived from isosorbide [130], 2,3:4,5-di-*O*-methylene-galactitol [131], 2,4:3,5-di-*O*-methylene-d-mannitol [131] and the bicyclic acetalized diol 2,4:3,5-di-*O*-methylene-d-glucitol (Glux) [132] have been incorporated into poly(butylene terephthalate) by SSM. Glux-based materials showed higher melting points and glass transition temperatures than the other sugar-based copolyesters prepared by SSM. These remarkable thermal properties were a direct result of the inherently rigid structure of Glux and the relatively slow randomization of the block-like chemical microstructure of the Glux-based copolyesters in the melt.

The relatively poor reactivity of the secondary hydroxyl groups at the 2- and 5-positions of isohexides is as a major drawback that limits their use as a polyester monomer [133]. Isoidide dicarboxylic acid (IIDCA) is a 1-carbon extended isohexide derivative that should have a higher reactivity with retention of rigidity [134]. In this way, a series of polyesters based on IIDCA and linear α,ω-diols HO–(CH_2_)*_n_*–OH (*n* = 2, 4, 6, 8, 10, 12) has been synthesized under rather mild reaction conditions. Weight-average molecular weights in the range of 13,000–34,000 g/mol were indicated and demonstrated a higher reactivity of IIDCA compared to the parent isohexides [135]. It should be stated that those polyesters based on ethylene glycol, 1,4-butanediol and 1,10-decanediol can be considered fully bio-based.

The bicyclic carbohydrate-based diol 2,4:3,5-di-*O*-methylene-*d*-mannitol (Manx) is highly reactive in polycondensation and capable of producing stereoregular polymers with fairly high molecular weights. This monomer has been employed to get aliphatic polyesters by reaction with dimethyl succinate. The homopolymer showed good properties and appears interesting for applications where biodegradability and molecular stiffness are priority requirements. In addition, random copolymers have been obtained using mixtures of the carbohydrate diol and 1,4-butanediol in the reaction with dimethyl succinate. All copolymers were semicrystalline, degraded enzymatically faster than PBS and displayed tunable thermal and mechanical properties according to the content in Manx units [136].

## 6. Degradation of Poly(alkylene dicarboxylate)s

**Figure 9.**
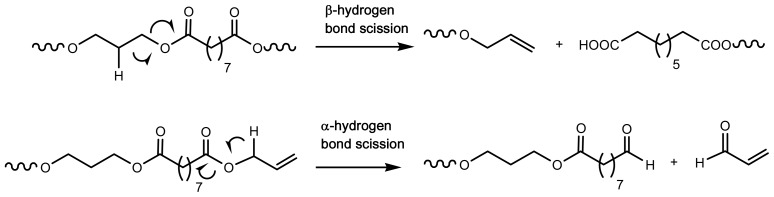
Schemes showing the β- and α-hydrogen bond scissions for aliphatic polyesters derived in this case from 1,3-propanediol and azelaic acid, based on [144].

Besides biodegradability, thermal stability and thermal degradation behavior of biodegradable polymers are important for their processing, application, and thermal recycling and in general are crucial for the development of safe materials. Therefore, thermal degradation of aliphatic polyesters based on dicarboxylic acids and diols has extensively been studied [137–144]. In general, these polyesters can be well-processed since start to decompose at temperatures significantly higher than their melting point. Decomposition has been described to take place, mainly, through β-hydrogen bond scission and secondarily by α-hydrogen bond scission (Figure 9). Thermal degradation under an oxidate environment may proceed according to a different mechanism and for example α-H abstraction mechanism that led to the formation of a hydroperoxide intermediate was characteristic of PBS thermal oxidation [138].

Aliphatic polyesters can easily be degraded enzymatically and constitute nowadays an important group of polymers to solve white pollution. Enzymatic degradation studies suggested that biodegradation rate was faster for polyesters made from moieties containing around six carbon atoms, whereas increasing or decreasing the spacing between ester groups made the polymers less susceptible to enzymatic degradation [145]. Thus, enzymatic hydrolysis of a series of poly (propylene alkane dicarboxylate)s with four to ten methylene units between the ester groups showed a maximum degradation rate for the suberate derivative and pointed out the significant role of the ester group density along the main chain [145].

Nevertheless, biodegradation rate is highly influenced by the degree of crystallinity, spherulite size and lamellar structure. Furthermore, melting temperature plays an important role since a polymer having a lower melting temperature is more susceptible to biodegradation due to the higher flexibility of their polymeric chains that allows fitting more easily into the active sites of enzymes [146].

Enzymatic degradation studies performed with high molecular weight poly(butylene succinate-*co*-ethylene succinate)s indicated that the degree of crystallinity was the dominant factor upon the degradation rate, whereas the sequence distribution of butylene and ethylene succinate subunits had little effect upon lipase activity [99].

Enzymatic degradation of network polyesters derived from polyhydric alcohols (*i.e.*, 1,1,1-trimethylolpropane, 1,2,3,4-butanetetrol, d-glucitol, glycerol and pentaerithritol) and aliphatic dicarboxylic acids containing different numbers of methylene groups (*i.e.*, 4, 6–14 and 20) has been studied [147–149]. Network structures are interesting since can provide better physical and chemical properties such as resistance to heat distortion and resistance to chemicals than linear polymers. Results obtained from series derived from the same dicarboxilic units indicated that degradation rate decreased with the increase in the number of hydroxyl groups in the alcohol units (*i.e.*, with the increase on the cross-linking density) [149]. Furthermore, a remarkable dependence of enzymatic degradation on methylene chain length was observed and specifically samples with shorter methylene chains were more resistant to degradation as a consequence of the difficulty for the enzyme to penetrate into samples with smaller network size.

Degradation of semicrystalline polymers must also occur in the crystalline phases, mainly during the later stages of decomposition despite the closely-packed crystalline regions hinder the enzymatic attack. Single lamellar crystals obtained by crystallization from dilute solutions are ideal systems to detect how enzymatic degradation proceeds. Extensive studies have been performed with single crystals of polyesters such as poly([R]-3-hydroxybutyrate) [150,151] poly(ɛ-caprolactone) [152] (PCL), poly(l-lactide) [153], poly(ethylene succinate) [154] and poly(tetramethylene adipate) [155]. Recognition of the position of ester bonds in the molecular chain is required by the active site of the enzyme for cleavage of the molecular chain and consequently it seems very important that these groups were accessible either on the folding surface or on the lateral faces of lamellar crystals.

**Figure 10.**
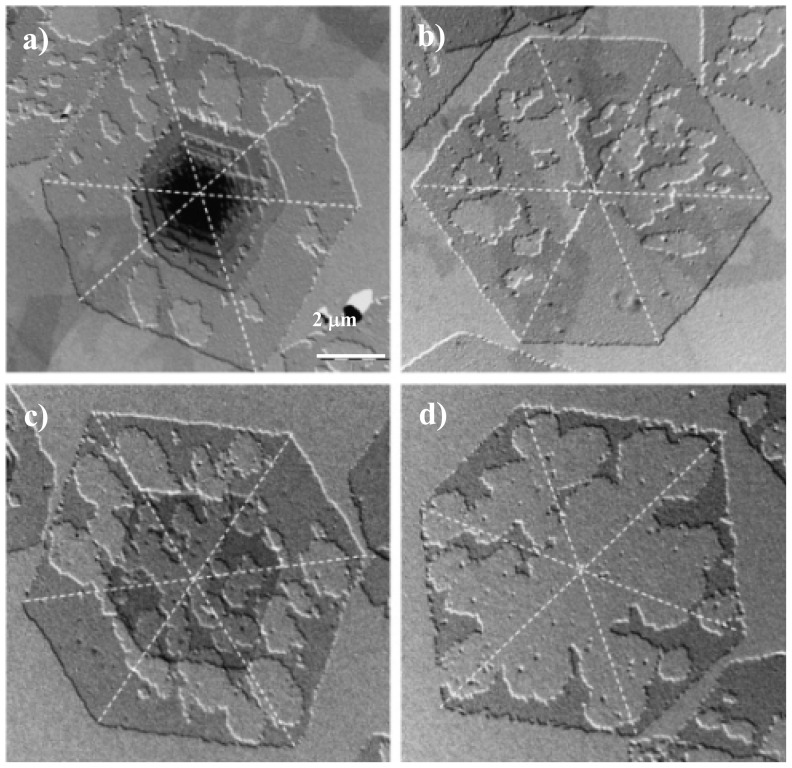
Lamellar crystals of poly(octamethylene suberate) after exposure to the enzymatic medium containing lipase for 2 (**a**); 2.5 (**b**); 3 (**c**) and 3.5 h (**d**). Reproduced from Casas and Puiggalí [156], copyright (2009), by permission of Elsevier Science Ltd.

Usually, enzymatic degradation progressed mainly from the edges of lamellar crystals without a decrease in molecular weight and lamellar thickness. However, the central portion of poly(butylene adipate) crystals was also degraded by enzymatic attacks, suggesting the existence of loosely-packed chain regions inside lamellae [155]. Enzymatic degradation studies performed with poly(octylene suberate) showed that in this case the main attack was on the lamellar surfaces [156]. Interestingly, crystal sectors had different susceptibility to enzymatic attack and furthermore degradation was observed to progress preferentially along a crystalline direction (Figure 10). It was concluded that both molecular packing and the nature of molecular folds play a crucial role in the enzymatic degradation process.

**Figure 11.**
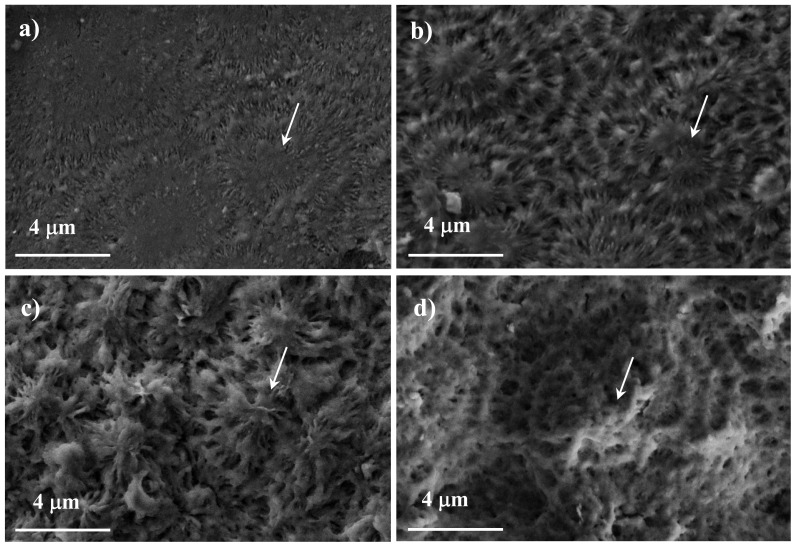
Scanning electron micrographs of a poly(butylene azelate-*co*-butylene succinate) sample after exposure to the lipase medium at 25 °C for 3 (**a**); 7 (**b**); 14 (**c**) and 21 days (**d**). White arrows point out spherulitic morphologies that are progressively highlighted. Reproduced from Díaz *et al.* [105], copyright (2014), by permission of Elsevier Science Ltd.

Enzymatic degradability of polyalkylene dicarboxylates derived from BDO and succinate or azelate units (*i.e.*, polyesters 4,4 and 4,9) differed considerably as presumable from their high different melting points. Samples having distinctive, controlled biodegradability could be easily generated by copolymerization. Hence, poly(butylene azelate-*co*-butylene succinate) copolymers had significant amorphous domains that facilitated enzymatic attack and led to post-exposure surface textures that were clearly different from those developed in the more crystalline homopolyester samples [105]. Constituent spherulites were highlighted during degradation when they had the appropriate size and were surrounded by significant amorphous zones. In a second step, degradation was observed to take place inside the spherulites and revealed a ringed structure. Edge-on and flat-on lamellae should have different susceptibility to the enzymatic attack and consequently it was possible to detect these ringed morphologies (Figure 11). Results pointed out the capability of enzymes to degrade crystalline regions in a way that depended on morphology (*i.e.*, flat-on or edge-on crystals) and the supramolecular arrangement.

Enzymatic degradation by immobilized lipase of PBS, its binary copolymers with ɛ-caprolactone or 1,4-cyclohexane dimethanol, and the corresponding ternary copolymers was evaluated in tetrahydrofurane/toluene (2:1 *v*/*v*) mixed organic solvent containing a small amount of water. The minimum and maximum degradation yields corresponded to PBS (40%) and the ternary copolymer (54%), respectively [157].

Biodegradation rate of PBS is relatively slow because of its high crystallinity, which furthermore can be increased during storage and service. In this sense incorporation of a third monomer (*i.e.*, a diol or a dicarboxylic acid) is an interesting alternative since may improve degradation properties and even extend its applications. Copolymers incorporating ethylene glycol [158,159] and propylene glycol [160–162] units have been studied, being found that the crystallization rate was dependent on the final composition. Similar features were found when BDO was polymerized with different ratios of dimethyl succinate and dimethyl adipate [163]. Nevertheless, it is expected a random microstructure for the derived copolymers that should lead to a sharply decrease on the melting temperature and consequently a limitation on the applied interest of the resulting samples [46]. Negative effects on both processability and mechanical properties must also be taken into account [164]. These problems can be avoided by preparing multiblock copolymers as those derived from the incorporation of poly(ethylene succinate) (PES) into PBS by using 1,6-hexamethylene diisocyanate (HDI) as a chain extender [165]. The melting point temperature (*T*_m_) and relative degree of crystallinity (*X*_c_) of the copolyesters were found to decrease first and then increase with PES content. The copolyesters showed excellent mechanical properties (e.g., fracture stress and strain of 61.8 MPa and 1173%, respectively, were determined for the copolymer having similar weight percentages of both blocks). Furthermore, degradation rate of these copolyesters was regulated in a wide range by their compositions, being the lowest rate found for the PBS homopolymer.

Studies on the biodegradation of aliphatic polyester nanocomposites show contradictory results about the influence of nanoparticles. On the one hand, an enhanced degradation is postulated mainly due the presence of hydroxyl groups in the added nanoparticles (e.g., the terminal hydroxylated edge groups of silicate layers) [166]. Basically, the hydrolysis of polyester/clay nanocomposites seems to depend upon both the nature of the pristine layered silicate and the surfactant used for the modification of the layered silicate [167]. Furthermore, the hydrophilicity of the silica surface enhanced the susceptibility of the nanocomposite to microbial attack as demonstrated for PBS/silica nanocomposites [168]. In the same way, hydrophilic fillers may enhance the degree of swelling of the otherwise hydrophobic matrix [169].

On the other hand, a decrease on the biodegradation rate has also been reported. In this case, arguments are based on the barrier properties of nanoparticles that should increase the difficulty for the micro-organism to reach the bulk matrix due to the more tortuous diffusion path [170]. Decrease on the biodegradation rate has also been related to interactions of the matrix with the nanofiller, water permeability, degree of crystallinity and anti-microbial property of the nano-filler [171].

The comparative effect on the enzymatic hydrolysis of three nanocomposites prepared by *in situ* polymerization and based on a poly(propylene sebacate) matrix and a 2 wt. % content of either fumed silica nanoparticles (SiO_2_), multiwalled carbon nanotubes (MWCNTs), or montmorillonite (MMT) has recently been reported [172]. A similar hydrolysis mechanism was determined, but in all cases nanoparticles hampered the action of the enzymes. It was postulated that the available surface area for hydrolysis was hindered and also that significant interactions took place between nanoparticles and the polymer matrix.

The addition of MWCNTs and MMT was found to enhance the thermal stability of the polymer, while SiO_2_ nanoparticles do not affect it [173]. The decomposition of nanocomposites proceeded with a complex reaction mechanism with the participation of at least two different steps: mainly through β-hydrogen bond scission and, secondarily, through α-hydrogen bond scission.

**Figure 12.**
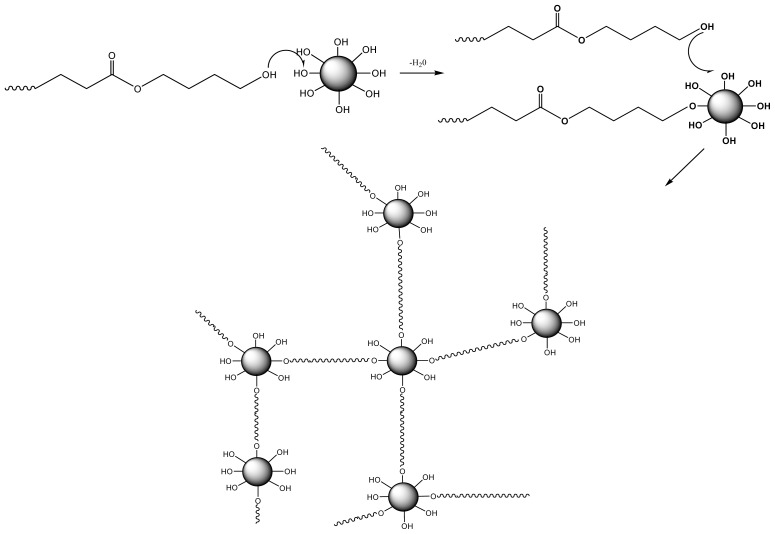
Cross-linked structures obtained by reaction between the hydroxyl end groups of PBS and the surface silanol groups of fumed silica nanoparticle.

Properties of PBS/SiO_2_ nanocomposites prepared by the *in situ* polymerization were carefully investigated to determine the potential interactions taking place between the hydroxyl end groups of PBS and the surface silanol groups of SiO_2_ [174]. It was found that at low concentrations the SiO_2_ nanoparticles acted as chain extenders, increasing the molecular weight of PBS, while at higher loadings they resulted in extended branching and crosslinking reactions (Figure 12), leading to smaller hydrodynamic dimensions and an apparent decrease on the molecular weight. Silica nanoparticles acted as nucleating agents, increasing the crystallization rate of PBSu, although the degree of crystallinity was slightly reduced.

## 7. Applications of Poly(alkylene dicarboxylate)s

**Figure 13.**
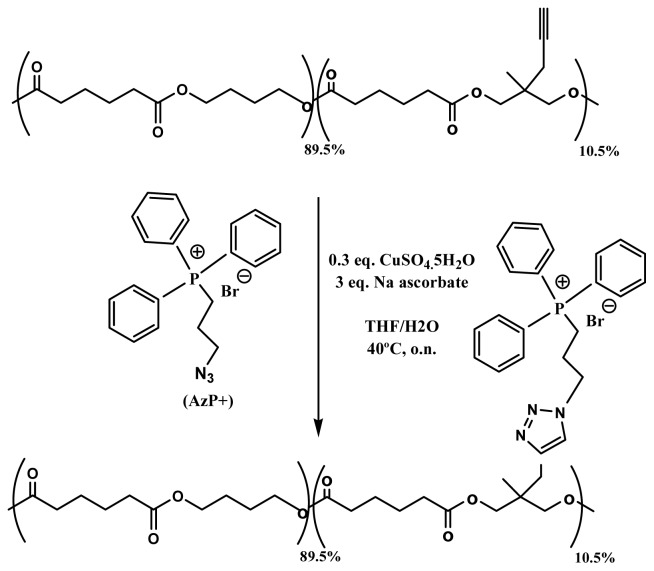
Synthetic of poly(butylene adipate) functionalized with quaternary phosphonium groups, based on [176]. The alkyne-containing polyester was prepared from 1,4-butanediol, the alkyne-containing diol 2-methyl-2-propargyl-1,3-propanediol and adipic acid.

Development of antimicrobial packaging films has logically interest in hospital environments where sterile conditions are required for the medical equipment, but also in the food industry in order to increase the safety of food products. Antimicrobial agents can be added to films and also chemically bounded to its surface. In the first case, activity is linked to the migration of the agent which can alter properties of the food product (e.g., taste and quality). This problem does not exist in the second case, but the lack of functional groups in the polymer chains and also the demanding reaction conditions become restrictive points that may limit its application. Functionalization of polyesters can be easily performed by copper catalyzed azide-alkyne “click” chemistry [175], a reaction that can be carried out under mild conditions that do not alter the chain length of the polyester backbone. In this way, alkyne-containing poly(butylene adipate) was effectively functionalized with a quaternary phosphonium group, known for their antimicrobial properties (Figure 13) [176]. The resulting polyester showed great antimicrobial activity through direct contact without freely available active groups and consequently with potential application for food or medical purposes.

**Figure 14.**
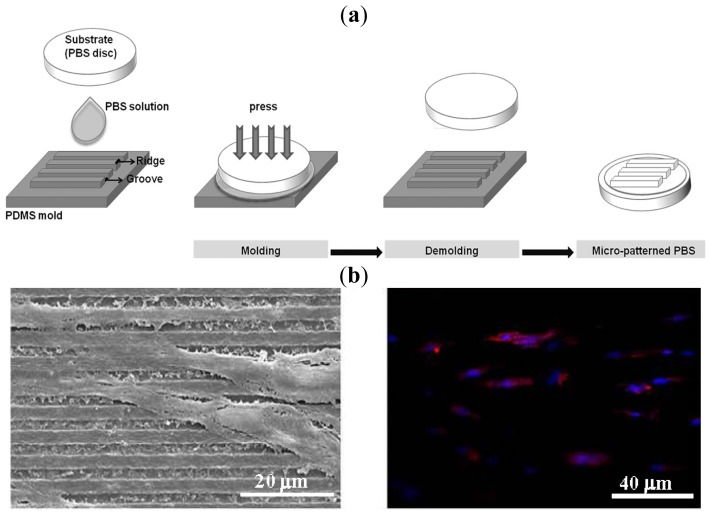
(**a**) Schematic representation of the preparation of micropatterned PBS surface. (**b**) SEM (left) and immunostaining (right) of human adipose stem cells (hASCs) cultured onto micropatterned PBS surfaces. Reprinted with permission from [180]. Copyright (2010), by permission of Elsevier Science Ltd.

The arrangement in specific architectures of micro- and nanoscale biological elements present in the extracellular matrix (ECM) becomes essential for normal tissue function. For example, we should mention the parallel array organization of fibroblasts and cardiomyocytes in native myocardial tissue that ensures adequate electrical properties or the alignment of collagen fibers in the bone that provides high tensile strength [177,178]. Therefore, tissue engineering strategies include the control of cellular alignment, which can be achieved by means of micropatterning techniques [179]. A simple method to control the alignment of human adipose stem cells (hASCs) over biodegradable PBS has recently been reported [180]. Specifically, a variety of micropatterned surfaces of PBS were microengineered by micromolding (Figure 14) and their *in vitro* biological behaviour evaluated. Results clearly demonstrated that the engineered surfaces were able to maintain a high viability and direct the orientation of seeded hASCs in contrast with the random orientation found on non-patterned surfaces.

Materials with low elastic modulus (*i.e.*, in the 0.1–100 MPa range) are preferred for soft tissue engineering applications, which is a limitation due to scarcity of biodegradable polymeric material possessing such elastomeric mechanical properties. In fact, scaffolds are designed to mimic the extracellular matrix of the target tissue, which usually is an elastic, resilient and highly hydrated polymer network. Efforts have mainly been focused to develop biodegradable polyurethanes, trimethylene carbonate-based copolymers and polyesters (ɛ-caprolactone- and sebacate-based polymers) [181–184].

Reactive blending appears as an efficient and easy method to get copolymers displaying modulated final properties. To this end, bioresorbable poly(butylene/diethylene glycol succinate) multiblock copolymers were specifically synthesized [185,186] starting from the parent homopolymers (*i.e.*, PBS and poly(diethylene glycol succinate) (PDGS)) and used to fabricate biomimetic electrospun scaffolds. Interestingly, new materials supported cell growth and had a more pronounced elastomeric behaviour and a faster degradation rate than PBS [187].

**Figure 15.**
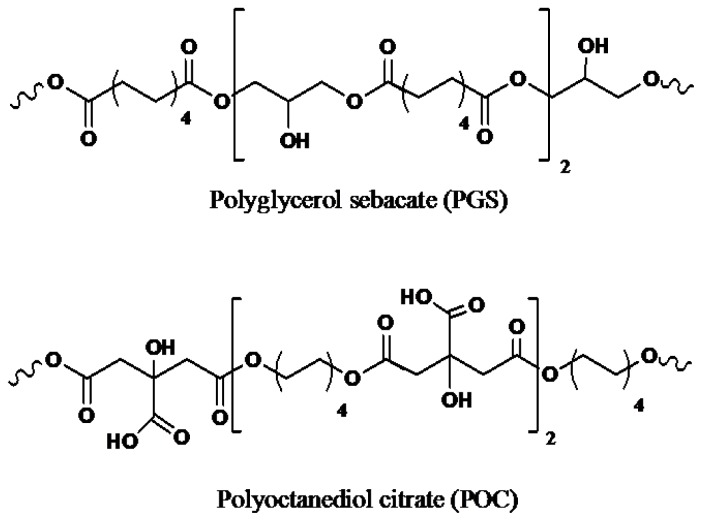
Chemical structures of linear poly(octanediol citrate) and poly(glycerol sebacate) with pendant hydroxyl and carboxyl groups.

Scaffolding materials have also been developed from elastomeric polyesters constituted by citric acid (CA) and sebacic acid (SA) [188–192]. These monomers are considered biocompatible since participate in different metabolic cycles in the human body [184,192–194] and can react with multifunctional alcohols (e.g., glycerol and octanediol) to yield hydrophilic and elastic hydrogels. Poly(octanediol citrate) (POC) and poly(glycerol sebacate) (PGS) (Figure 15) are polyesters with pendant –COOH and –OH functional groups that brings hydrophilicity and allows also the formation of physical crosslinks between polymer chains that are characteristic of elastomeric materials. Interestingly, PGS and POC have been employed to get biphasic systems ideal for blood vessel tissue engineering where PGS is used as an inner non-porous phase and POC constitutes the porous outer layer [195].

Random poly[octanediol-*co*-(citric acid)-*co*-(sebacic acid)] copolymers have also been considered [196]. These elastomeric materials exhibit versatility in mechanical properties, hydration and hydrolytic degradation by varying the citric acid/sebacic acid concentration in the reaction medium. Furthermore, cell culture results clearly showed that simple alteration of the copolymer composition influenced cellular growth on the polymer surface, a feature advantageous to expand the repertoire of available biodegradable polyesters suitable for bone tissue engineering.

Rai *et al.* have recently been reported an excellent review concerning design and fabrication (e.g., rapid prototyping, solid-free form fabrication, micromolding, microablation and electrospinning) and biomedical applications (e.g., hard to soft tissue engineering, controlled drug delivery and tissue adhesives) of PGS based devices [197].

Sebacic acid has also been reacted with multifunctional alcohols giving rise to materials with good properties for cell growth and control over physicochemical and degradation properties through the use of different polyols [195].

Tissue engineering scaffolds can be easily prepared by the electrospinning technique, which is able to produce nanofibers from a wide range of polymers [198,199]. Furthermore, many kinds of drugs can be incorporated into such nanofibrous mats and then successfully released from them without a significant loss of their activity. Different examples can be mentioned about the specific use of electrospinning to render scaffolds from poly(alkylene dicarboxylate)s.

First attempts to get biodegradable PBS nanofibers were reported by Jeong *et al.* when different solvent mixtures were assayed [200]. PBS fibers had average diameters in the range of 125–315 nm and were highly crystalline.

Electrospun 1,6-diisocyanatohexane-extended PBS fiber mats appeared suitable as bone scaffolds since supported the grown and proliferation of bone cells [201]. A novel PBS/wollastonite/apatite composite scaffold useful for bone tissue applications was fabricated via electrospinning and biomimetic processes. The microstructure of these scaffolds could be adjusted by controlling the wollastonite content and the incubation time in simulated body fluid [202].

Reactive blending of PBS and poly(diethylene glycol succinate) rendered multiblock bioresorbable copolyesters with an elastomeric behaviour and able to be processed as electrospun scaffolds. These were found to support the growth and preserve the cardiac phenotype markers of cardiomyocyte H9c2 cells, demonstrating its potential utility in soft tissue engineering applications [187].

Biodegradable PBS fiber mats containing silver nanoparticles were prepared by the electrospinning process. Bacterial growth was found to be inhibited for a long period of time due to the long-term release performance of Ag from these fiber mats [203].

Drug-loaded PBS microspheres useful for wound treating were prepared by electrospinning. Diameters of these microspheres could be controlled within the 5 to 25 μm range [204].

Non-linearly elastic biomaterials were successfully fabricated from poly(glycerol sebacate) (PGS) and PLLA using the core/shell electrospinning technique. PLLA was used as the shell material to constrain PGS during the curing process and was also added into the PGS-prepolymer core solution in order to increase the viscosity to suitable values for the electrospinning process [205]. These fibrous materials possessed excellent biocompatibility, supported human stem-cell-derived cardiomyocytes over several weeks in culture and had comparable properties than muscle tissue. Hence materials appear interesting for the therapy of soft tissues exposed to cyclic deformation [206]. PGS/gelatin core/shell nanofibers have also been produced by electrospinning for cardiac tissue engineering applications [207].

Core-shell fibrous scaffolds are usually designed with a core material that dictates the mechanical properties, and a shell polymer that solely affects the cell functions. Although the coaxial process seems ideal to combine properties of different materials, the control of the desired ratio between the two polymers may be difficult. Hence, electrospining of a solution containing the elastomeric polymer (*i.e.*, PGS) and a fairly rigid biomaterial appears a simple and interesting alternative. Specifically, aligned nanofibrous PGS:gelatin scaffolds were fabricated, being demonstrated that they supported cardiac cell organization, phenotype and contraction and could potentially be used to develop clinically relevant constructs for cardiac tissue engineering [208].

**Figure 16.**
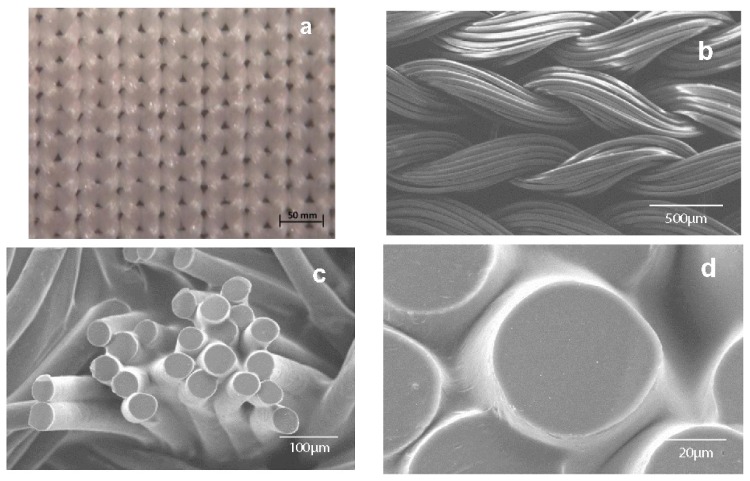
Morphology of PBS knitted constructs showing different levels of detail: Macroscopic image (**a**); top view (**b**) and fibre cross-sections (**c**,**d**). Reproduced from Almeida *et al.* [209], copyright (2013), by permission of Elsevier Science Ltd.

Knitted structures of synthetic or biological materials have been proposed to construct functional 3-D scaffolds applicable in the repair/replacement and regeneration of tissues. Knitted textile substrates may show better mechanical properties (e.g., extensibility and compliance) compared with other woven substrates due to highly ordered arrangement of interlocking loops. Knitting technology may offer superior control over the scaffold design (e.g., size, shape, porosity and fibre alignment), manufacturing and reproducibility. In this way, fiber-based finely tuned porous architectures have been produced from PBS (Figure 16), which has the advantage of having a high level of processing control, from the filament to the final textile structure [209].

Hydrolytically degradable copolyester biomaterials based on glycolic acid in combination with sebacic acid and ethylene glycol have also been studied taking into account that several sebacic acid-based polymers have been approved by the Food and Drug Administration (FDA) in biomedical applications [210]. Furthermore, sebacic acid is a highly suitable monomer for the preparation of polyesters since short-chain aliphatic acids tend to give intramolecular condensation reactions. The new hydrolytically degradable copolyesters were soluble in common organic solvents, opposite to poly(glycolic acid), being postulated good perspectives for biomedical applications such as tissue engineering scaffolds and drug release [211].

## 8. Poly(ester amide)s Derived from the Incorporation of Natural α-Amino Acids to Polyalkylene Dicarboxylates

An important limitation in the use of biodegradable (bioresorbable) polymers as biomedical materials is the potential toxicity of the degradation products, and so, research towards synthetic biodegradable polymers has mainly focused on materials entirely composed of naturally occurring and nontoxic (“physiological”) building blocks. Among the physiological building blocks to be used for constructing biodegradable biomaterials naturally occurring α-amino acids (AA) are one of the most attractive ones due to an ample availability and versatile nature—many amino acids are produced in many thousand tones [212] and the global market is forecast to reach US$ 11.6 billion by the year 2015 [213], and almost all of 20 naturally occurring AA can be used for constructing biodegradable biomaterials that allows to tune their properties in the widest range. The AA that represent hetero-bifunctional compounds, allow to incorporate two the most desirable hetero-links into the polymeric backbones—ester bond via *C*-terminus (carboxyl group) and *H*-bond forming links such as amide bond via *N*-terminus (amino group). The former, well discussed in the paragraphs above, provides reasonable biodegradation (hydrolysis) rates of the polymers, improves processability and decreases immunogenicity of AA-based polymers; the latter provides desired mechanical properties at lower molecular weights, increases hydrophilicity of the polymers and promotes their active interaction with the surrounding tissues in a desirable manner after implantation. All these impart to poly(ester amide)s (PEA) an obvious advantage over biodegradable polyesters [214]. It is reasonable to note here that pure polyamides made of AA—poly(α-amino acid)s have been proved to be less suitable as biodegradable materials for biomedical engineering use for many reasons, such as low rate of biodegradations and poor processability [215].

**Figure 17.**
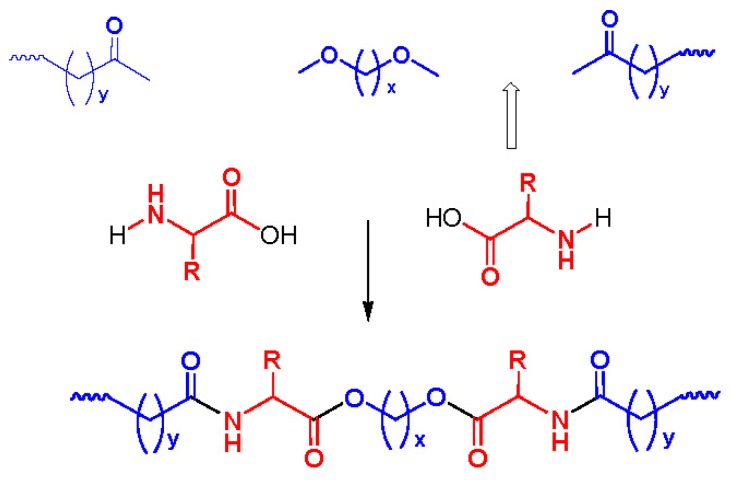
Schematic representation of AABBP as products of the insertion (empty arrows) of AA between ester bonds.

There are three the most important types of biodegradable PEA composed of AA—polydepsipeptides that are the co-polymers of α-hydroxy (mostly glycolic and lactic acids) and α-amino acids [216–218], tyrosine dipeptide based polymers [219], and polymers composed of AA and other nontoxic building blocks such as fatty diols and dicarboxylic acids [217,218,220]. Only the latter type of PEA, called as Amino acid based biodegradable poly(ester amide)s (AABBP) [220], relates to the family of alkylene dicarboxylates and will be discussed below in more details. The AABBP (Figure 17) can be considered as derivatives of alkylene dicarboxylates (AA–BB type polyesters) that are obtained by the insertion of AA residues amongst carbonyl group and ether oxygen in polyesters.

**Figure 18.**
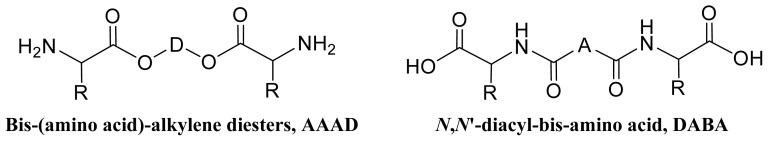
Two types of AA based monomers—bis-nucleophilic AAAD and bis-electrophilic DABA. R, side chain of AA; D, diol residue; A, Diacid residue.

This kind of AABBP can be prepared using two types of dimerized forms of AA as key monomers (Figure 18), either bis-(amino acid)-alkylene diesters (AAAD) or *N*,*N′*-diacyl-bis-amino acids (DABA).

The former contain two desirable ester bonds and the latter two desirable amide bonds per molecules. The synthesis and some specific features of these monomers are discussed in brief below.

### 8.1. Monomers for Synthesizing AABBP

#### 8.1.1. AA Based Monomers

One of the key monomers for synthesizing AABBP are bis-nucleophilic AAAD, which are in fact diamino-diesters. These compounds are stable in the salt form, commonly as di-*p*-toluenesulfonic acid (TosOH) salts (labeled as AAAD-S). One of the most rational ways of preparing AAAD-S is direct condensation of AA (2 mol) with fatty diols (1 mol) in refluxed benzene or toluene in the presence of TosOH monohydrate (2 mol). The TosOH·H_2_O serves as both the reaction catalyst and amino group protector, preventing undesirable side reactions of amino groups with inherent ester groups of AAAD that lead to the formation of diketopiperazines, other cyclic and low-molecular-weight products of unknown structure (See Ref. [221] and references cited therein).

**Figure 19.**
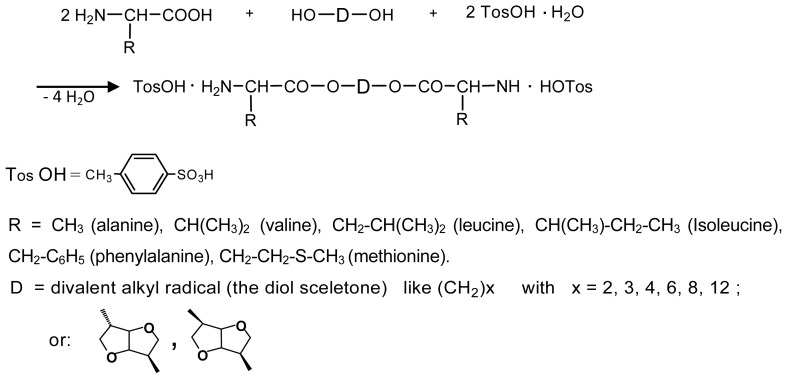
Synthesis of AAAD-S on the basis of hydrophobic AA.

Two moles (plus a slight excess that serves as a catalyst) of TosOH·H_2_O are needed for the synthesis of AAAD-S on the basis of hydrophobic AA that proceeds according to Figure 19.

**Figure 20.**
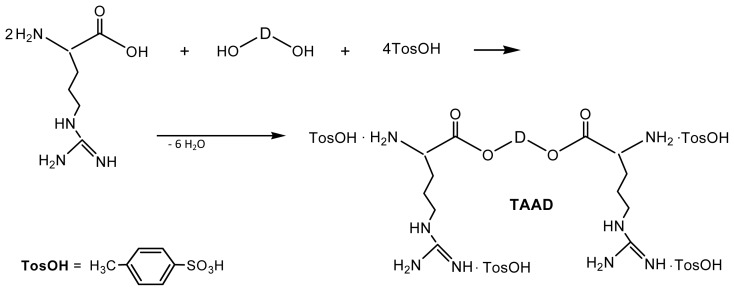
Synthesis of AAAD-S on the basis of l-arginine. D = (CH_2_)_x_ with *x* = 2, 3, 6, 12 or (CH_2_)_2_–[O–(CH_2_)_2_]_k_ with *k* = 1, 2, 3, 5, 11.

For the synthesis of diamino-diester monomers on the basis of amino acid l-arginine four moles (plus a slight excess that serves as a catalyst) of TosOH·H_2_O should be used since two moles are consumed by the strong basic guanidine groups on the l-arginine side chain. As a result tetra-*p*-tolue-nesulfonic acid salts are obtained, as it is demonstrated in Figure 20.

**Figure 21.**
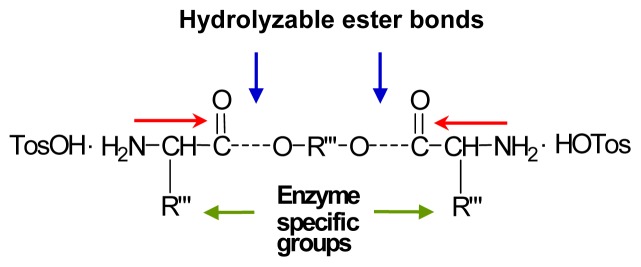
Structural peculiarities of AAAD-S monomers. Blue arrows, ester bonds; green arrows, enzyme specific groups; red arrows, the nonconventional “head-to-head” orientation.

This synthetic strategy allows to generate diamino-diester monomer with two inherent biodegradable (hydrolyzable) ester bonds (blue arrows), two enzyme specific groups (green arrows), and the nonconventional “head-to-head” orientation (red arrows) (Figure 21) of AA put at a monomer stage, that provides low immunogenicity of the goal AABBP [220]. The desirable H-bond forming amide bonds are formed at a propagation stage after the interaction of α-amino groups of AAAD-S with active derivatives of dicarboxylic acids (To be discussed below).

The merits of the synthetic schemes above consist in direct condensation of free amino acids with diols in refluxed benzene or toluene that results in the monomers with 80%–95% yield most of which are purified by recrystallization from water that makes the technology highly cost-effective. A wide variety of alkylenediols including α,ω-polymethylenediols and biobased diols such as dianhydrohexitols [217,218,220,222,223], as well as oligo-ethylene glycols [224–227] were used for synthesizing bis-amino-acid based diester salt monomers AAAD-S depicted in Figures 19 and 20. A huge variety of AA and diols used for synthesizing diamino-diester salt monomers allows varying material properties of the AABBP made of them.

**Figure 22.**
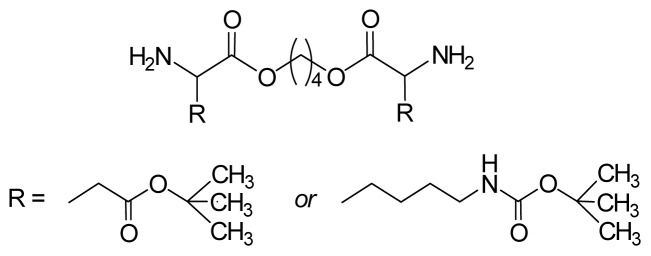
AAAD monomers derivatives of β-*C* protected and ɛ-*N* protected lysine.

Interesting AAAD (free bases) promising as starting monomers for synthesizing functional AABBP were obtained by the group of Mequanint and Gillies [228,229] following to multistage synthetic strategy widespread in peptide chemistry: they used heterodiprotected polyfunctional amino acids (purchasable products)—*N*-CBZ-l-aspartic acid-*β-t*-butyl ester and *N*^α^-CBZ-*N*^ɛ^-*t*-BOC-l-lysine, condensed (esterified) these compounds with 1,4-butanediol using coupling agent (DCC), and selectively removed one of the functional groups (another one was deprotected in polymers, see Figures 28 and 30 below). The structures of these functional monomers are represented in Figure 22.

The AAAD above were successfully used as comonomers of AAAD-S (made of hydrophobic AA) in both solution active and interfacial polycondensations for obtaining functional polymers with satisfactory molecular weights. No data, however, were reported on the synthesis of high-molecular-weight homo-AABBP on their basis presumably due to insufficient purity of these complex and nontrivial monomers (they were not subjected to purification after synthesis—deprotection of corresponding precursors) that might be farther decreased due to side reactions typical for alkyl esters of α-amino acids as free basis (discussed in a brief above). The decreasing molecular weight of AABBP with increasing the mole fraction of functionalized monomers in comonomer pairs AAAD/AAAD-S [228] could speak for this assumption.

Another type of AA-based monomers is DABA in which the desirable amide bonds are incorporated at a monomer stage, and the biodegradable (hydrolysable) ester bonds are formed at a propagation stage. In these monomers the orientation of AA is also nonconventional, “tail-to-tail” type that can diminish the immunogenicity of the goal AABBP.

**Figure 23.**
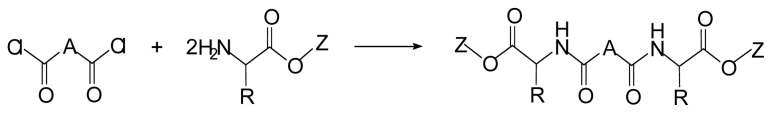
Synthesis of DABA (Z = H) and DABA-M (Z = CH_3_). A = (CH_2_)_4_ or *p*-C_6_H_4_; Z = H or CH_3_; For R, see Figure 19 above.

The DABA are obtained by the interaction of two moles of AA with diacid chlorides under Shotten-Baumann reaction conditions or in dioxane using NaOH, KOH or MgO as an HCl acceptor [230,231], according to Figure 23.

For synthesizing AABBP, DABA are used mostly as active bis-electrophilic monomers such as bis-azlactones [231,232] or activated diesters [233] that were obtained via appropriate transformations of DABA, or dimethyl diesters (DABA-M in Figure 23) that were synthesized by Puiggalí *et al.* [234] via interaction of diacid chlorides with AA (glycine) methyl ester with diacid chlorides in chloroform in the presence of triethylamine as HCl acceptor.

DABA as polycondensation type monomers can reveal a dual nature, either bis-electrophilic (when carboxyl groups are transformed into active derivatives) or bis-nucleophilic (when carboxyl groups are transformed into salts). In both cases the biodegradable (hydrolysable) ester bonds are formed at a chain propagation stage that will be discussed below.

#### 8.1.2. Monomers—Counter-Partners for Synthesizing AABBP

Active derivatives of dicarboxylic acids—either activated diesters or diacid chlorides were used as bis-electrophilic monomers in polycondensation reactions with AAAD/AAAD-S. Fatty diols were used as bis-nucleophilic monomers in polycondensation reactions with bis-azlactones or DABA-M, and bis-electrophilic dihalo-alkanes are used as bis-electrophilic monomers in polycondensation reaction with the salts of DABA.

Most of diacid chlorides and fatty diols are purchasable products. Activated diesters of general formula R_1_–O–CO–A–CO–O–R_1_ are obtained using three synthetic methods: (i) by interaction of diacid chlorides Cl–CO–A–CO–Cl with various hydroxyl compounds HOR_1_ (activating agents), or by direct interaction of dicarboxylic acids (ii) with HOR_1_ in the presence of various condensing (coupling) agents or (iii) with various *trans*-esterifying agents that are derivatives of HOR_1_. All three methods give activated diesters in a good yield ranged from 60% to 90% (See [220] and references cited therein). The application of activated diesters allows to obtain AABBP on the basis of short-chain dicarboxylic acids such as succinic and adipic acids, dichlorides of which are less stable hydrolytically and, hence, less suitable for synthesizing high-molecular-weight polymers via interfacial polyconden-ation, or on the basis of such diacids, dichlorides of which are not available at all due to instability towards chlorinating agents. It is worth mentioning here that in case of succinyl chloride the hydrolysis is not the only factor resulting in low-molecular-weight polymers—the fast cyclization of intermediate amide into five-membered succinimide cycle leads to undesirable chain termination as well. When using more steady activated di-succinates as bis-electrophilic monomers, high-molecular-weight film-forming polysuccinamides were synthesized [235].

### 8.2. AABBP Made of AAAD Monomers

#### 8.2.1. AABBP via Solution Active Polycondensation

This method is based on the application of various activated diesters as bis-electrophilic monomers in polycondensation reaction with bis-nucleophiles, mostly diamines [236,237]. The activated diesters showed much more “calm temper” as compared with diacid chlorides which are traditionally used in solution or interfacial polycondensations [238]. A large variety of activating agents HOR_1_ allows the tuning of the diesters activity in a wide range [236,237]. Besides, the activated diesters are stable against both amide type solvents and tertiary amines under the conditions of solution polycondensation [239] that minimizes undesirable site reactions and results in the formation of high-molecular-weight polymers. This stability of activated diesters allows the use of salts of diamines such as AAAD-S in solution polyamidation reactions, which needs tertiary amines for deprotonation of primary amino groups that are necessary for their interaction with electrophile-activated ester groups resulting in amide bond formation and representing the chain propagation reaction. Mostly di-*p*-nitrophenyl esters of diacids (HOR_1_ = *p*-nitrophenol) are used for synthesizing amide-type polymers due to the facile synthesis via all three methods above, a high reactivity and vast availability and low price of p-nitrophenol. It is, however, rather problematic to remove *p*-nitrophenol from the polymers after polycondensation by simple washing with water due to its poor solubility in water. From this point of view more promising look bis-*N*-oxysuccinimidyl esters of diacids that also result in high-molecular-weight polyamides [240], and a by-product of polycondensation; *N*-hydroxysuccinimide is easily soluble in water.

##### Regular AABBP and Related Polymers

To this category we refer the polymers that have no functional groups except two terminal functional groups, normally one nucleohpilic and one electrophilic. According to the polycondensation theory of Kricheldorf [241,242] a substantial portion of molecules obtained via polycondensation have no terminal functional groups at all since they form macrocycles.

**Figure 24.**
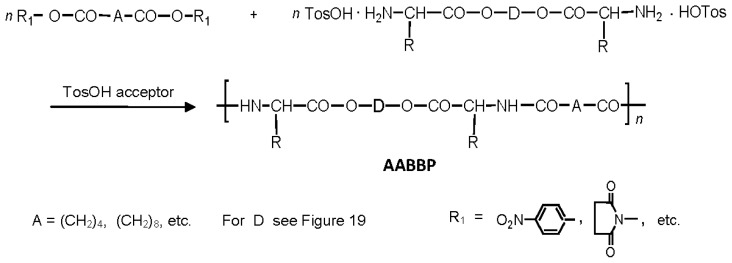
Synthesis of AABBP via active polycondensation.

The first regular AABBP were synthesized by interaction of AAAD-S with activated diesters R_1_–O–CO–A–CO–O–R_1_ of α,ω-alkylenedicarboxylic acids [A = (CH_2_)_y_] in organic solvents [243–246], according to Figure 24.

The polycondensation is normally carried out in high-polar aprotic solvents like *N*,*N*-dimethyl formamide (DMF), *N*,*N*-dimethyl acetamide (DMA), *N*-methyl-2-pyrrolidone (*N*-MP), dimethyl sulfoxide (DMSO) and chloroform [246]. Since the alcoholysis of activated diesters is much slower compared to the aminolysis [247], protic solvents (alcohols) can also be used as reaction media for synthesizing AABBP. For example, a high-molecular-weight film-forming polymer was synthesized by polycondensation of di-*p*-nitrophenyladipate with AAAD-S composed of l-leucine and 1,6-hexanediol in isopropyl alcohol [248]. Tertiary amines (mostly triethylamine) are typically used as *p*-toluenesulfonic acid acceptor though inorganic acceptors such as sodium or potassium carbonates are also effective [246].

**Figure 25.**
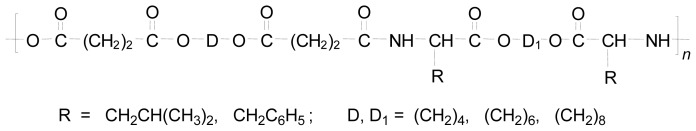
AABBP composed of alkylene disuccinates.

Solution active polycondensation allowed synthesizing high-molecular-weight polymers on the basis of monomeric alkylene dicarboxylates, namely alkylene disuccinates (diester-diacids) that were synthesized by interaction of diols (1 mol) with succinic anhydride (2 mol). The diester-diacids were transformed into activated di-*p*-nitrophenyl esters that were used in Figure 24 as bis-electrophilic monomers resulting in high-molecular-weight polysuccinates [249], represented in Figure 25.

The polymers contain two additional ester bonds (in total four ester bonds) per elemental link and showed increased biodegradation rates. Additionally, the enhanced hydrolysis of polysuccinates is linked with intramolecular catalysis (see [235] and references cited therein).

**Figure 26.**
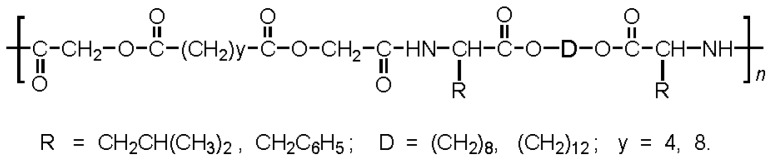
AA-BB type polydepsipeptides.

Another representatives of AABBP containing four hydrolyzable ester bonds per elemental link are polymers composed of *O*,*O′*-diacyl-bis-glycolic acids (another kind of diester-diacids) obtained by interaction of glycolic acid (2 mol) with diacid chlorides (1 mol) [250,251]. The diester-diacids were transformed into activated di-*p*-nitrophenyl esters (like alkylene disuccinates above) that were used in Figure 24 as bis-electrophilic monomers resulted in a high-molecular-weight AA-BB-type polydepsipeptides, represented in Figure 26.

The regular AABBP synthesized via solution active polycondensation have high molecular weights (*M*_W_ ≤ 180,000 Da, GPC) and rather narrow polydispersity (1.20–1.81). The polymers have a wide range of glass transition temperature (*T*_g_ = 5–102 °C), some of them are semicrystalline with *T*_m_ = 103–153 °C. The highest *T*_g_ showed the polymers composed of bicyclic diols, dianhydrohexitols. The chemical structure influences the mechanical properties of AABBP which varies in a wide range: tensile strength from 15–20 to 80–100 MPa, elongation at break from up to 200%, and Young’s modulus up to 2 GPa. The AABBP are soluble in common organic solvents such as DMF, THF, methylene chloride, chloroform, some of them in dioxane, acetone, and alcohols (methanol, ethanol, isopropanol) (See [220] and references cited therein). The low melting temperatures and solubility in common solvents make AABBP easily processable into different shapes.

##### Functional AABBP

**Figure 27.**
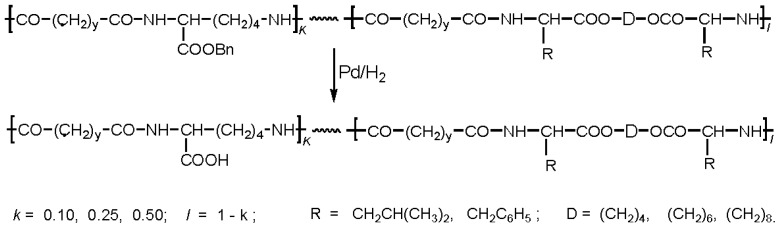
Synthesis of AABBP with lateral carboxyl groups.

The solution active polycondensation turned out especially useful for synthesizing AABBP containing various functional groups in lateral chains. The first representatives of AABBP with lateral carboxyl groups (in fact polyanions) were obtained by Jokhadze *et al.* [252] using l-lysine derivative as a diamine comonomer, according to Figure 27 below.

These polymers showed excellent biocompatibility [253,254] and were successfully used as vascular stent coatings [255].

**Figure 28.**
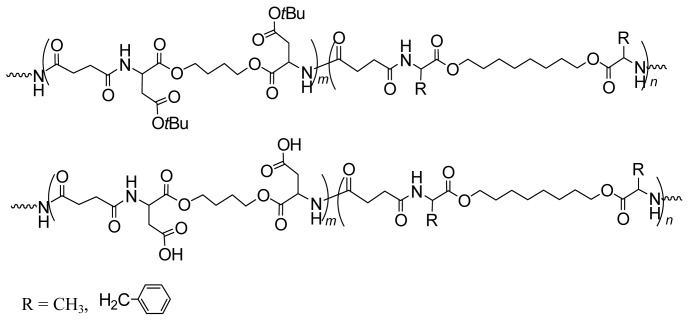
*co*-AABBP-polyacids composed of bis-(l-aspartic acid)-1,4-butylene diester.

The polyacids depicted in Figure 27 represent copolymers of poly(ester amide)s and polyamides. Pure AABBP-polyacids (copolymers) were prepared by the group of Mequanint and Gillies [229] via solution active polycondensation of comonomer pairs bis-(l-aspartic acid-β*-t*-butyl ester)-1,4-butylene diester (Figure 22) and AAAD-S (composed of l-alanine and l-phenylalanine) with di-*p*-nitrophenyl succinate, and subsequent deprotection of the intermediated *C*-protected AABBP, as depicted in Figure 28.

The polyacids were further functionalized by transforming the lateral COOH groups into activated *N*-oxysuccinimidyl esters [229] that are promising biodegradable carriers for the conjugation of active molecules such as drugs, targeting groups, cell signaling molecules, *etc.*

**Figure 29.**
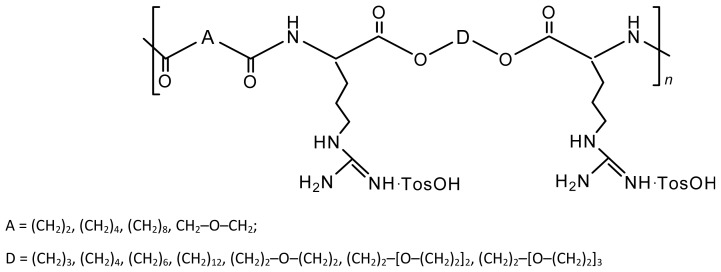
Cationic AABBP composed of l-arginine.

Polycationic AABBP with lateral guanidine groups, represented in Figure 29, were obtained by solution active polycondensation of arginine-based AAAD-S according to Figure 20 with activated diesters of succinic, adipic, sebacic [225–227,256] or diglycolic acids [227]. The polymers showed outstanding cell compatibility and are promising non-viral gene delivery agents.

**Figure 30.**
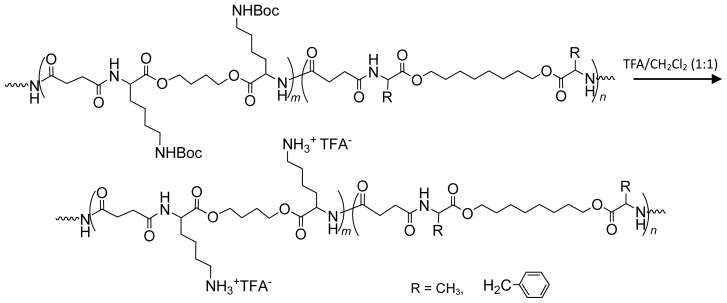
Cationic AABBP composed of bis-(l-lysine)-1,4-butylene diester.

Another types of cationic AABBP (copolymers) containing primary amino groups in the lateral chains were prepared by solution active polycondensation of comonomer pairs bis-(*N*^ɛ^-*t*-BOC-l-lysine)-1,4-butylene diester (Figure 22) and AAAD-S (composed of l-alanine and l-phenylalanine) with di-*p*-nitrophenyl succinate, and subsequent deprotection of the intermediated *N*-protected AABBP [229,257], as depicted in Figure 30.

**Figure 31.**
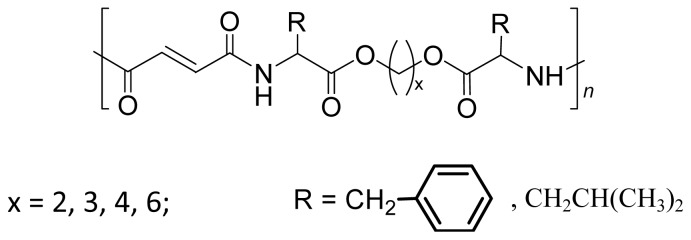
Unsaturated AABBP composed of fumaric acid.

A series of unsaturated AABBP, depicted in Figure 31, were synthesized by solution polycondensation of activated di-*p*-nitropheyl fumarate with AAAD-S [258–260]. The polymers can be used as biodegradable carriers for covalent attachment biochemicals through sulfhydryl or amino groups, or to transform into various functional polymers: polyols, polyacids or polyamines by interaction with corresponding thiol or amino reagents; the former gave a much higher degree of transformation (90%–95% *vs.* 23%–24%) [260].

**Figure 32.**
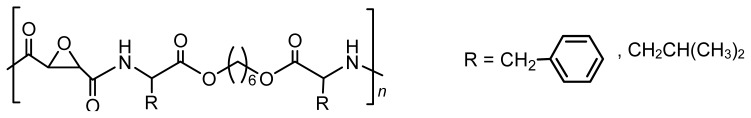
AABBP composed of epoxy-succinic acid.

Another functional, “ready-for-use” type epoxy-AABBP, depicted in Figure 32, were synthesized by Zavradashvili *et al.* [261] via solution polycondensation of activated di*-p-*nitrophenyl-*trans*-epoxy succinate with AAAD-S.

High-molecular-weight polymers (*M*_W_ up to 76,000) with desirable material properties have been obtained. Epoxy-AABBP could chemically be modified further under mild conditions: oxirane groups along the backbone were reacted with both nucleophilic and electrophilic reagents, as well as subjected to thermal curing. The macromolecular transformations of the epoxy-polymers substantially broaden material properties and, hence, the potential to apply AABBP as absorbable drug carriers and surgical devices.

**Figure 33.**
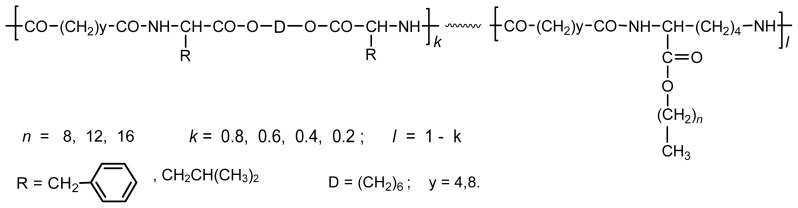
Brush-like copolymeric AABBP composed of l-lysine *n*-alkyl esters.

Copolymeric AABBP with a brush-like architecture (Figure 33) containing long-chain alkyl substituents were obtained by Katsarava and coworkers [262,263] using l-lysine *n-*alkyl esters as comonomer in a mixture with AAAD in solution active polycondensation.

The polymers are promising biodegradable drug carriers that can be drug-loaded via hydrophobic forces. The polymers form nanocomplexes with PEG [263] that are also of interest as drug-carriers.

Various AABBP containing free OH groups were obtained by Gomurashvili *et al.* [264] by solution polycondensation of AAAD-S composed of unsubstituted AA glycine and glycerol with di-*p*- nitrophenyl esters of succinic, glutaric, adipic, and diglycolic acids. Depending on the synthetic strategy used, three types of hydroxyl-containing polymers were synthesized: (i) with pending primary hydroxyls; (ii) with pending secondary hydroxyls; or (iii) a copolymer containing both primary and secondary glycerol hydroxyls (not shown here). Polymers composed of short aliphatic diacids such as succinic, glutaric, and diglycolic acids were water soluble.

**Figure 34.**
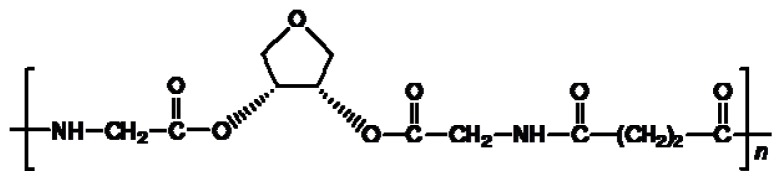
Water-soluble poly(ester amide)s (PEAs) on the basis of 1,4-anhydroerythritol, glycine, and succinic acid.

Water-soluble AABBP, having the structure given in Figure 34, were also obtained by polycondensation of AAAD-S composed of 1,4-anhydroerythritol and glycine with di-*p*-nitrophenyl succinate [264].

**Figure 35.**
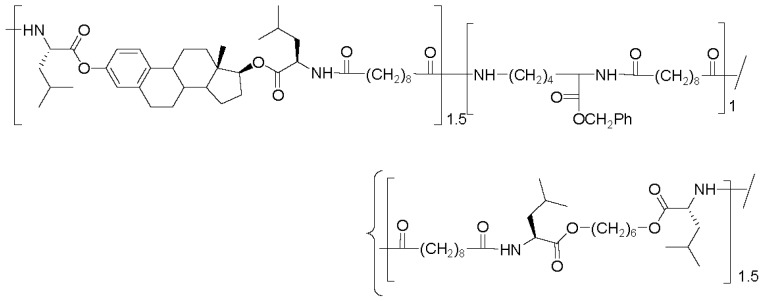
Drug-containing AABBP composed of 17-β-estradiol, 1,6-hexanediol, l-leucine, l-lysine benzyl ester, and sebacic acid.

The strategy of the synthesis of AABBP via solution active polycondensation allows constructing biodegradable polymeric drugs. Therapeutic copolymer (Figure 35) was obtained by Gomurashvili *et al.* [255] by polycondensation of di-*p-*nitrophenyl sebacate with three comonomers: two AAAD-S composed of l-leucine/1,6-hexanediol and l-leucine/17-β-estradiol, and di-*p*-toluensulfonic acid salt of l-lysine benzyl ester.

#### 8.2.2. AABBP via Interfacial Polycondensation

**Figure 36.**
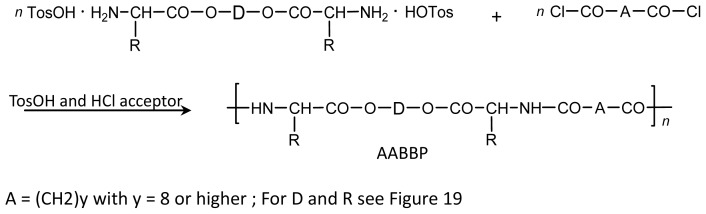
Synthesis of AABBP via interfacial polycondensation.

The interfacial polycondensation in a two phase system water/hydrophobic organic solvent is well known and widely used method of polymer synthesis [238]. The main advantage of this method consists of simplicity and a high rate of the reaction. The general scheme of the synthesis of AABBP via interfacial polycondensation is outlined in Figure 36.

Puiggalí *et al.* [234,265–271] successfully applied this method to the synthesis of various AABBP composed of hydrophobic AA. They obtained high-molecular-weight polymers showing good film-forming properties by interaction of AAAD with sebacoyl chloride. Mequanint *et al.* [257,272] used the same method for synthesizing AABBP including functional ones (copolymeric AABBP) depicted in Figures 28 and 30. This team carried out a comparative study of solution active and interfacial polycondensations [257] and showed that the latter led to the AABBP with higher yields and molecular weights (Table 1). The interfacial polycondensation, however, results in high-molecular-weight polymers when hydrophobic diacid chlorides such as sebacoyl chloride or higher diacid chlorides are used due to the hydrolytic instability of lower diacid chlorides. This is an important limitation of this method. Solution active polycondensation is applicable virtually to all dicarboxylic acids and represent more universal synthetic method.

### 8.3. AABBP Made of DABA-Based Monomers

#### 8.3.1. AABBP via Thermal and Biocatalytic Polycondensation

**Figure 37.**
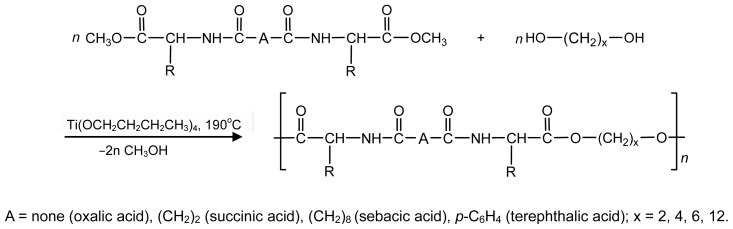
Synthesis of AABBP via thermal polycondensation.

High-molecular-weight AABBP were synthesized by Puiggalí and co-workers [234,271] via thermal polycondensation (polyesterification) of DABA-M with diols, as indicated in Figure 37.

This method was applied for synthesizing AABBP composed of unsubstituted amino acid glycine. This alternative synthesis route was undertaken because of the difficulty in esterifying 1,4-butanediol with glycine, *i.e.*, unavailability of AABBP composed of the said building blocks through solution active or interfacial polycondensations. The polyesterification was carried out in melt at 190 °C using titanium butoxide as a catalyst. Later on the method was applied to other diols such as 1,2-ethanediol, 1,6-hexanediol, and 1,12-dodecanediol though their esterification with glycine was not problematic.

Turnell *et al.* [273] used thermal polycondensation for synthesizing proline-based AABBP via interaction of corresponding DABA-M with diols. Polymers having molecular weights in the range from about 14,000 Da to about 77,000 Da were obtained. These proline-based AABBP assemble as nano-particles in aqueous solutions and form complexes with various cations and biologics, including hydrophobic small molecule drugs and biologics.

Besides the successful synthesis of AABBP unavailable via solution or interfacial polycondensation discussed above (e.g., AABBP composed of 1,4-butanediol and glycine), the advantages of the thermal polycondensation *vs.* interfacial polycondensation are as follows: (1) oxalic acid derivatives can be obtained with high viscosity, a fact that is not possible by interfacial polymerization because of the high hydrolysis rate of the oxaloyl dichloride; and (2) the intrinsic viscosities of polymers synthesized by thermal condensation are usually higher, especially when interfacial polymerization implies unstable diacid chlorides such as succinyl chloride or the said oxalyl chloride (Table 2). The synthesis of AABBP via thermal polycondensation, however, has some limitations: (1) the synthesis with ethylene glycol was unsuccessful because a high degree of decomposition at high temperature; (2) polymers on the basis of succinic acid are of lower viscosity compared to sebacic acid derivatives, presumably due to the cyclization (imidization) at high temperature. In addition, some polymers such as those containing oxaloyl or terephthaloyl residues showed some coloration.

In general, film- (from the melt state or from the solution) and fiber-forming properties were found for polymers with an intrinsic viscosity higher than 0.5 dL/g [234]. Potentially, the method of thermal polycondensation could be applied to the DABA-M on the basis of other hydrophobic amino acids since *T*_g_ and *T*_m_ of corresponding AABBP [246] are far below of polyesterification temperature (190 °C). When applicable, the technological advantage of thermal polycondensation consists in the possibility to process polymers from melt directly after the polycondensation, that is without the separation and purification of the resulting polymers. This makes the method rather promising from the industrial point of view.

Monomer pairs DABA-M/alkylene diols are also promising for enzyme catalyzed synthesis of AABBP. In a preliminary study of Omay and Katsarava [274] polymer with *M*_W_ up to 13 KDa was obtained after polycondensation of dimethyl ester of *N*,*N′*-bis-sebacoyl-l-leucine with 1,12-dodecanediol in the presence of Novozym-435.

#### 8.3.2. AABBP via Azlactone Method

**Figure 38.**
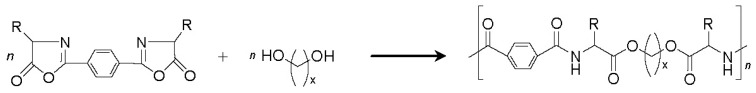
Synthesis of AABBP on the basis of bis-azlactone.

AABBP composed of aromatic terephthalic acid were obtained by interaction of corresponding bis-azlactones with diols according to Figure 38. Cleaver and Pratt [275] first described the poly(ester amide) polymer obtained by interaction of leucine-based bis-azlactone (R = CH_2_CH(CH_3_)_2_ in Figure 38) with 1,6-hexanediol (*x* = 6) in chloroform. Only low-molecular weight polymer (inherent viscosity *η*_inh_ = 0.20 in m-cresol at 25 °C and *c* = 0.1%) was obtained. Neither acid nor base type catalyst led to the polymer of higher molecular weight.

Kharadze *et al.* [232] carried out the polymerization reaction of phenylalanine-based bis-azlactone (R = CH_2_–C_6_H_5_ in Figure 38) with 1,4-butanediol (*x* = 4) in various organic solvents in the presence of acid catalysts. The best result (yield 96%, reduced viscosity *η*_red._ = 0.40 dL/g in *m*-cresol at 25 °C and *c* = 0.5 g/dL) was achieved in *O*-dichlorobenzene at 160 °C in the presence of trifluoroacetic acid.

#### 8.3.3. AABBP via Nucleophilic Substitution (NS)

**Figure 39.**
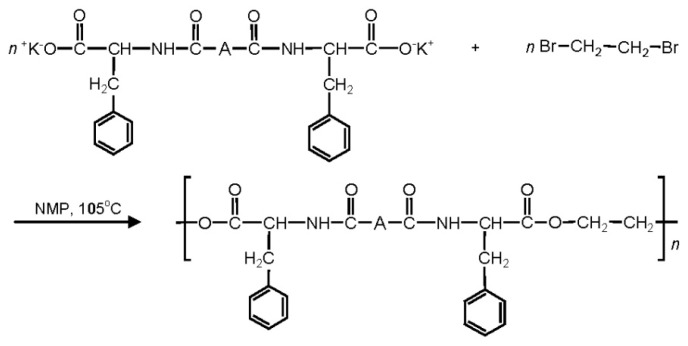
Synthesis of AABBP via nucleophilic substitution (NS) polycondensation.

As noted above DABA can be used also as bis-nucleophilic monomers by transforming into corresponding salts. Potassium salts of DABA (composed phenylalanine and adipic or terephthalic acid) were used in solution polycondensation with 1,2-dibromoethane as it is outlined in Figure 39.

AABBP with reduced viscosity up to 0.28 dL/g (*m*-cresol, 25 °C, *c* = 0.5 g/dL) were obtained after this NS polycondensation reaction [232].

#### 8.4. **AABBP** Made by Combining AAAD-S and DABA Monomers

**Figure 40.**
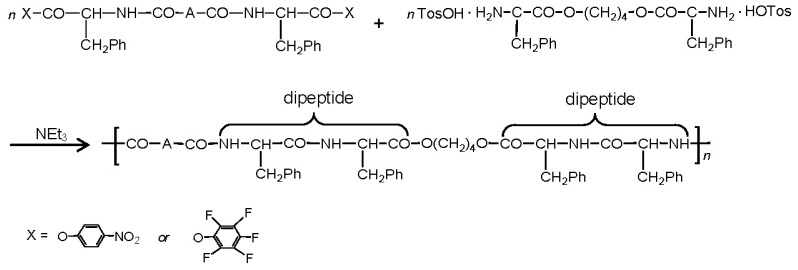
Synthesis of dipeptide-containing AABBP.

There are only a few samples of the synthesis of AABBP by the combination of both types of AA-based monomers: AAAD-S and DABA. Activated di-*p*-nitrophenyl esters of two DABA composed of phenylalanine and adipic or terephthalic acids [233] were polycondensed with AAAD-S composed of phenylalanine and 1,4-butanediol in hexamethylphosphoramide, as represented in Figure 40.

Relatively low-molecular-weight AABBP with *η*_red._ = 0.19–0.32 dL/g (*m*-cresol, 25 °C, *c* = 0.5 g/dL) and 86%–92% yield, containing dipeptide Phe-Phe fragments inserted amongst carbonyl group and ether oxygen instead of AA in Figure 17, were obtained.

**Figure 41.**
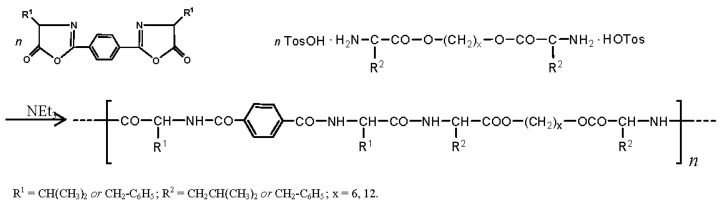
Synthesis of dipeptide-containing AABBP via bis-azlactones.

**Figure 42.**
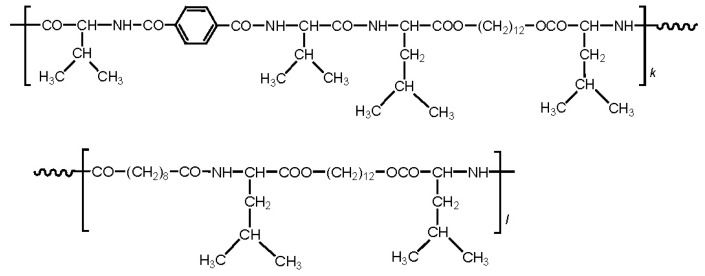
Copolymeric AABBP on the basis of azlactone and activated sebacate and AAAD-S.

Very recently this kind of AABBP was synthesized by solution polycondensation of valine and phenylalanine based bis-azlactone (R^1^ = CH(CH_3_)_2_ and CH_2_–C_6_H_5_, accordingly) with AAAD-S using triethylamine as *p*-toluenesulfonic acid acceptor [276], according to Figure 41. Low-molecular-weight powdery polymers with *η*_red._ = 0.1–0.2 dL/g (in 1,1,2,2-tetrachloroetane/phenol (3:1) mixture at 25 °C and *c* = 0.5 g/dL) were obtained that can be explained by the presence of triethylamine that catalyzes side reactions of azlactones [275]) resulting in chain termination. To diminish these side reactions bis-azlactones in part were substituted by activated diester (di-*p*-nitrophenyl sebacate) that increased molecular weight of the obtained *co*-AABBP (*η*_red._ = 0.91 dL/g at *k*/*l* = 30/70) [277], depicted in Figure 42.

The azlactone method is a convenient way for incorporating rigid and hydrophobic terephthalic acid residue into the AABBP backbones. The incorporation of terephthalic acid fragments substantially decreases the stickiness of AABBP and renders them suitable for preparing resuspendable micro and nanoparticles—promising vehicles for drug delivery.

### 8.5. Biocompatibility and Some Applications of **AABBP**

AABBP composed of adipic acid, l-phenylalanine, and 1,4-butanediol supported the growth of human osteosarcoma and fibroblasts cells and showed the material to be biocompatible [220]. Elastomeric functional *co*-AABBP composed of sebacic acid (1.00 mol), l-leucine (1.50 mol) and 1,6-hexanediol (0.75 mol), and l-lysine (0.25 mol) [252] showed excellent blood and tissue compatibility in both *in vitro* [253] and *in vivo* (pigs) [254] tests. The same polymer selectively supported the *in vitro* growth of epithelial cells [253]. The *in vivo* biocompatibility was tested in porcine coronary arteries, comparing the polymer-coated stents with bare metal stents in 10 pigs [254]. All animals survived till sacrificed 28 days later. Prior to sacrifice, angiography revealed identical diameter stenosis in both groups. Histology confirmed similar injury scores, inflammatory reaction, and area stenosis. These results showed the polymer has a high potential for cardiovascular applications.

Recently Yamanouchi *et al.* [256] reported that the arginine-based AABBP showed good cell compatibility over a wide range of dosages and had minimal adverse effects on the cell morphology, viability, and apoptosis. Memanishvili *et al.* [225,227] showed that arginine-based AABBP having PEG-like polymeric backbones, possess higher cell compatibility than the said arginine-based polymers [256] not containing ether bonds.

The above-mentioned biological studies of several AABBP indicate that this family of biodegradable polymers is biocompatible.

**Figure 43.**
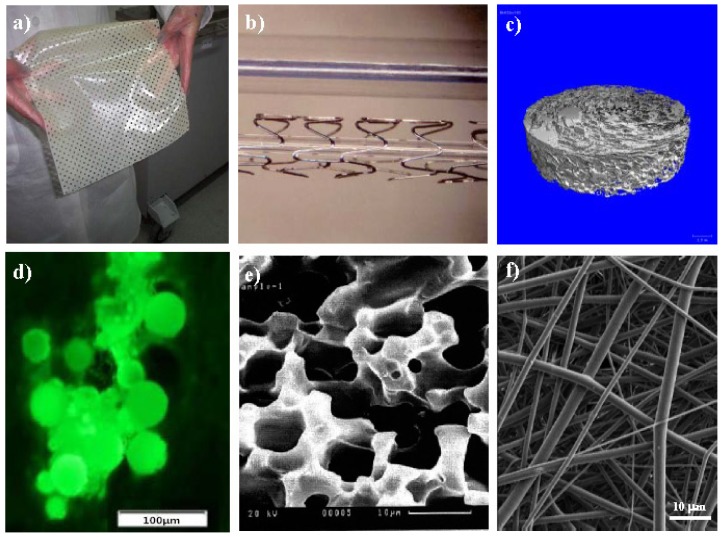
Some biomedical applications of AABBP as resorbable biomaterials: (**a**) an artificial skin PhagoBioDerm; (**b**) drug eluting vascular stent; (**c**) bone substitute; (**d**) microspheres for drug delivery; (**e**) porous scaffolds for cell cloning; (**f**) electrospun microfibers.

Selected representatives of AABBP were used for constructing biodegradable hydrogels, nanoformulations, drug-eluting devises and coatings, and so forth. Guo *et al.* used the unsaturated AABBP for obtaining hybrid hydrogels through photochemical conjugation with PEG-diacrylate [278]. The biodegradable hybrid hydrogels are promising for many biomedical and pharmaceutical applications, such as drug delivery systems, tissue engineering, *etc.* Legashvili *et al.* [263] used brush-like *co-*AABBP (Figure 33) to obtain molecular complexes with PEG that are promising as nanocarriers of drugs. Yamanouchi *et al.* [256] evaluated complexation of a novel family of synthetic biodegradable l-arginine-based AABBP with DNA, for their capability to transfect rat vascular smooth muscle cells, a major cell type participating in vascular diseases. In whole, Arg-based AABBP [225–227,256] can be attractive candidates for non-viral gene carriers owning to their high cellular uptake nature and reliable cellular biocompatibility. Katsarava *et al.* [279] used AABBP and their blends for constructing various medical biocomposites. One of them, registered as “PhagoBioDerm” in Republic of Georgia, is produced as elastic films (Figure 43a) and represents innovative wound-dressing device (artificial skin) consisting of lytic bacteriophages, antibiotics, pain killer, and proteolytic enzymes. PhagoBioDerm showed an excellent therapeutic effect in the management of infected wounds and ulcers (of both trophic and diabetic origin) and in the complex treatment of infected local radiation injuries caused by the exposure to ^90^Sr. This team developed also bactericidal wound dressing that represents an alcohol solution of biodegradable *co*-AABBP containing antimicrobials. The preparation sprayed onto the wound forms a thin, elastic, and transparent film that accelerates healing of superficial wounds, ulcers, and burns (See [220] and references cited therein).

Elastomeric functional *co*-AABBP [252] of a high biocompatibility [253,254] revealed high elastic properties and excellent adhesion to stainless steel, and was used by MediVas, LLC (San Diego, CA, USA) for developing drug-eluting vascular stent coating (Figure 43b). Currently the polymer-coated stents are under clinical trials. In general, the *co-*AABBP and related polymers are considered as promising for innovative drug delivery technology (MediVas polymer technology was licensed to DSM Biomedical, http://www.dsm.com/en_US/html/dbm/homepage.htm) [280].

Trollsas *et al.* of Abbott Vascular [281] studied series of AABBP with various chemical structures, specifically synthesized to optimize the everolimus release rate and the mechanical integrity of drug eluting stent coatings. This team showed that AABBP can be designed to have outstanding coating and controlled release properties that can potentially be used for complex medical application such as a drug eluting stent coatings, *etc.*

Some of AABBP show good mechanical characteristics and are promising as resorbable bone substitutes (Figure 43c). The polymers form also microspheres (Figure 43d) suitable as resorbable containers for drug delivery purposes, porous materials (Figure 43e) promising as scaffolds for cell cloning, and nanofibers (Figure 43f), including medicated ones, electrospun from non-toxic solvents like ethanol and apt as dressing for accelerated wound healing. The AABBP can also be made as viscos-flow mass (not shown) that could be impregnated with drugs and bioactive fillers and used, e.g., to seal bone cavities and regenerate/reconstruct bone tissues.

## 9. Conclusions

Poly(alkylene dicarboxylate)s constitute a family of biodegradable polymers with increasing interest since typical limitations associated to limited molecular weights have been overcomed by different improvements on synthesis procedures. Thus, polycondensation reactions have been performed by employing nonspecific enzymes, efficient catalysts such as triflates and chain extenders. Even cyclic ester oligomers have been used to get poly(alkylene dicarboxylate)s via ring opening polymerization. In general, these polymers have an additional interest since can be prepared from biobased monomers such as diols like BDO, fatty acids and other dicarboxylic acids like SA and carbohydrates. The disponibility of different monomers allows getting materials with a wide range of properties and applications. Thus, biodegradability can be tuned through the degree of crystallinity and even the melting point of samples, being fundamental the preparation of copolymers. Applications can involve rigid materials that are being developed through the incorporation of cyclic units and elastomeric materials that can mainly be pepared from multifunctional alcohols.

Incorporation of α-amino acids into a polyalkylene dicarboxylate chain gives rise to a series of biodegradable poly(ester amide)s with promising expectations since amide groups are able to establish strong intermolecular hydrogen bonding interactions and improve the limited thermal and mechanical properties of polyesters. In addition, polymer properties can also be varied according to the characteristics of the side group of the selected amino acid. The high versatility and biocompatibility provided by natural α-amino acids merit attention together with the fact that some polymers can be considered fully derived from renewable resources. Nowadays, a wide diversity of polymerization procedures have been successfully developed to get amino acid based biodegradable poly(ester amide)s (AABBP) with high yield and appropriate molecular weights (*i.e.*, from solution active, interfacial, thermal and biocatalytic polycondensations).

Current developments on poly(alkylene dicarboxylate)s and AABBP cover a broad spectrum that mainly concerns to biodegradable and biocompatible elastomers, hyperbranched polymers, functional polymers for conjugating drugs or cell signaling molecules, hydrogels, nonviral delivery vectors, scaffolds with multiple functionalities, stent coatings, and bactericidal wound dressings. Despite these advances, efforts appear still necessary to validate the promising properties and even to improve peformance characteristics of new AABBP.

## Figures and Tables

**Table 1.** Comparison of solution and interfacial polycondensation of AABBP (according to [257]).

PEA	Yield (%)	*M*_W_ (Da)	*M*_n_ (Da)	PDI
8-Ala-8-Sol	67	51,400	36,600	1.40
8-Ala-8-Int	68	62,500	45,100	1.39
8-Phe-4-Sol	85	103,000	53,500	1.93
8-Phe-4-Int	78	168,000	63,600	2.64
8-Phe-8-Sol	61	63,300	44,400	1.43
8-Phe-8-Int	60	111,000	71,800	1.55

**Table 2.** Comparison between the intrinsic viscosities [*η*] of AABBP according to the polycondensation method (according to Ref. [234]).

Polymer a	[*η*] b (dL/g)	

	Interfacial Polycondensation	Thermal Polycondensation
PGHGT	0.68	0.74
PGDGT	0.60	0.68
PGHG0	c	0.60
PGDG0	c	0.73
PGHG2	0.23	0.38
PGDG2	0.20	0.40
PGHG8	0.37	0.51
PGDG8	0.41	0.48

a Abbreviations: P, polymer; G, glycine; H, 1,6-hexanediol; D, 1,12-dodecanediol; T, terephthalic acid; 0, oxalic acid; 2, succinic acid; 8, sebacic acid;

b In dichlorocetic acid at 25 °C;

c No polymer obtained.
